# The trend analysis of HIV and other sexually transmitted infections among women of childbearing aged 15 to 49 years from 1990 to 2021 and its forecasting from 2022 to 2030

**DOI:** 10.3389/fpubh.2025.1702289

**Published:** 2025-12-19

**Authors:** Yingying Lin, Yilan Lin, Yongbiao Chen, Ziwei Xie, Xinyu Zhou, Xuemin Xiao, Xiaohong Wang

**Affiliations:** 1 First Clinical Medical College, Fujian University of Traditional Chinese Medicine, Fuzhou, Fujian, China; 2 Department of Gynecology, People's Hospital Affiliated of Fujian University of Traditional Chinese Medicine, Fuzhou, Fujian, China; 3 Xiamen Center for Disease Control and Prevention, Xiamen, Fujian, China; 4 Department of Dermatology, Fujian Medical University Union Hospital, Fuzhou, Fujian, China

**Keywords:** AIDS, sexually transmitted infection, women of childbearing age, global burden of disease, disability-adjusted life years

## Abstract

**Background:**

HIV and other sexually transmitted infections (STIs) among women of childbearing age need in-depth research because of the physiological characteristics and their disadvantages in obtaining equal health rights. We intend to present the burden of disease worldwide from 1990 to 2021 using data from the Global Burden of Disease (GBD) study and forecast the DALYs from 2022 to 2030.

**Methods:**

Using the 2021 GBD study, we researched the average annual percentage change (AAPC) of HIV and other STIs in incidence, prevalence, disability-adjusted life years (DALYs) rates for women of childbearing age aged 15 to 49 years across different age groups, sociodemographic index (SDI) regions, and nations. We used statistical Autoregressive Integrated Moving Average (ARIMA) models to forecast.

**Results:**

In 2021, there were 670,804 new AIDS cases in women of childbearing age globally, with a decrease in age-standardized incidence rate (AAPC −1.94, −2.21 to −1.67) and an increase in age-standardized DALYs rate (AAPC 1.67, 1.20 to 2.14) from 1990 to 2021. There were 286,514,651 new other STIst cases with an increase in age-standardized incidence rate (AAPC 0.12, 0.09 to 0.14) and a decrease in age-standardized DALYs rate (AAPC −0.15, −0.18 to −0.11). The largest increase in age-standardized incidence rate of HIV occurred in Oceania from 1990 to 2021. The SDI trends were the opposite of age-standardized incidence rate and age-standardized DALYs rate for HIV and other STIs among women of childbearingage. The STI and HIV epidemics are interdependent. Women aged 30–34 years were the group with the highest incidence and DALY rates. Trichomoniasis and chlamydia account for the majority of cases of other STIs. The DALYs rate of HIV showed a downward trend, and the DALYs rate of other STIs showed an upward trend from 2022 to 2030.

**Conclusion:**

Our study investigated the status of HIV and other STIs in women of childbearing age. The DALYs rate of HIV increased. The incidence rate of other STIs raised and the number of cases was so high that the harm could not be ignored. All sectors of the global community should pay attention to the prevention and control of STIs among women of childbearing age.

## Introduction

HIV infection remains a critical global public health challenge, posing a severe threat to human health and life. Since the onset of the AIDS epidemic, tens of millions of lives have been lost to AIDS-related causes, and approximately 40 million individuals currently live with HIV1. In 2023, the global median HIV prevalence among adults aged 15–49 was 0.7%, with 1.3 million new infections recorded (1). Certain populations exhibit disproportionately higher prevalence rates, including women and girls, as well as high-risk groups such as sex workers, men who have sex with men, transgender individuals, injecting drug users, and incarcerated persons. Notably, women and girls accounted for 44% of new HIV infections worldwide in 2023 (2). Sexually transmitted infections (STIs) significantly impact global health and reproductive well-being, contributing to psychological, social, and economic burdens that diminish quality of life.

This study aims to examine the global prevalence of HIV and other STIs—including trichomoniasis, gonorrhea, syphilis, genital herpes, and chlamydia—among women of reproductive age (15–49 years) and to identify key influencing factors. The findings are expected to inform targeted interventions for mitigating HIV and STI transmission, ultimately advancing women’s health and contributing to the broader goal of HIV eradication.

## Methods

### Study population and data collection

Data were sourced from the Global Burden of Disease, Injury, and Risk Factors Study (GBD) 2021 *via* the Global Health Data Exchange (GHDx) outcome tool. GBD 2021 provides comprehensive estimates of the global burden of 369 diseases and injuries across 21 regions, characterized by comparable geographic and epidemiological profiles, spanning the period from 1990 to 2021. This study focused on HIV and five STIs—chlamydial infection, genital herpes, gonococcal infection, syphilis, and trichomoniasis—based on data availability within GBD 2021, which exclusively reported burden estimates for these infections (3).

STI data in GBD 2021 were derived from multiple primary sources, including case notifications, cross-sectional studies, prenatal and community surveillance data, inpatient hospital records, health insurance claims, and datasets contributed by the GBD collaborator network. To minimize bias, populations at elevated risk for HIV and STIs—such as sex workers, men who have sex with men, and individuals living with HIV—were excluded from the analysis. HIV-related data sources in GBD 2021, accessible through the GHDx data input sources tool, included household seroprevalence surveys, national surveillance data (e.g., prenatal care records and health service reports), HIV incidence and prevalence estimates, intervention coverage data reported to UNAIDS (including antiretroviral therapy [ART] utilization, prevention of vertical transmission, and ART eligibility), GBD demographic inputs, and vital registration datasets.

Estimates of incidence, prevalence, and disability-adjusted life years (DALYs) for HIV/AIDS and STIs were extracted from GBD 2021 to assess trends at both global and regional levels among women of reproductive age. Data were further stratified by age group and socio-demographic index (SDI). The 15–49 age range was categorized into seven five-year brackets based on GBD classification. SDI, a composite measure of health-related social and economic factors, integrates the total fertility rate among women under 25, the mean years of education among individuals aged 15 and older, and lag-adjusted per capita income. Regions were classified into five SDI quintiles: low, low-middle, middle, high-middle, and high.

The Autoregressive Integrated Moving Average (ARIMA) model was used to predict the DALYs rate of HIV and other STIs worldwide (4). The model effectively captures patterns in time series data by integrating three key components: autoregressive (AR), differential (I), and moving average (MA). Its parameters are denoted as p, d, and q, which represent the autoregressive order, difference degree, and moving average order, respectively, and these parameters are estimated by Maximum Likelihood Estimation, using autocorrelation function (ACF) and the partial autocorrelation function (PACF) to determine the optimal values of p and q. The Akaike information criterion (AIC) and Bayesian information criterion (BIC) were used to select the optimal ARIMA model, the goodness of fit of the model was evaluated by MAPE (Mean Absolute Percentage Error) and RMSE (Root Mean Square Error). $\text{MAPE}=\frac{1}{n}\sum_{i=1}^{n}|\frac{y_{i}-{\hat{y}}_{i}}{y_{i}}|\times 100\%,\text{RMSE}=\sqrt{\frac{1}{n}}\sum_{i=1}^{n}{\left(y_{i}-{\hat{y}}_{i}\right)}^{2}$ , *n*: total number of observations; y_i_: observed value at the *i*-th time point (actual value); ${\hat{y}}_{i}$ : the forecast value at the *i*-th time point.

### Data analysis

Age-standardized incidence, prevalence, and disability-adjusted life years (DALYs) for HIV and other STIs among women of reproductive age (15–49 years) were estimated using the direct method of age standardization. This approach models prevalence as a weighted sum of independent Poisson-distributed variables. Standardized indicators were employed to quantify variations in the burden of HIV/AIDS and other STIs across different time periods and regions, with all rates expressed per 100,000 individuals. The 95% uncertainty intervals (UIs) were computed using the “ageadjust.direct” function from the epitools package in R.

Temporal trends in age-standardized HIV/AIDS incidence at global, regional, and national levels were analyzed using Joinpoint regression. This method detects inflection points in trends, segments the data accordingly, and calculates the annual percentage change (APC) with corresponding 95% confidence intervals (CIs) to assess epidemiological trajectories. Additionally, the average annual percentage change (AAPC), a weighted mean of APC values across segmented intervals, was derived to determine whether incidence rates exhibited an increasing, decreasing, or stable trend over a fixed period. Statistical analyses were performed using the Joinpoint Regression Program (version 5.0.2) and R (version 4.3.2). A two-tailed *p*-value < 0.05 was considered statistically significant.

## Results

### Global analysis

In 2021, an estimated 670,804 (95% CI: 596,594–762,161) new HIV cases were reported among women of reproductive age (15–49 years), corresponding to an age-standardized incidence rate of 34.7 cases per 100,000 population (95% CI: 34.70–34.70) (Table 1). Between 1990 and 2021, the global age-standardized incidence rate of HIV exhibited a declining trend, with an average annual percentage change (AAPC) of −1.94% (95% CI: −2.21 to −1.67). The burden of HIV, as measured by disability-adjusted life years (DALYs), reached 16,378,796 (95% CI: 14,479,032–18,796,594) in 2021, with an age-standardized DALY rate of 829.8 per 100,000 population (95% CI: 829.70–829.80). Despite the decline in incidence, the age-standardized DALY rate of HIV demonstrated an upward trend, increasing annually by 1.67% (95% CI: 1.20–2.14) over the same period.

**Table 1:** Number of cases, age-standardized rate (ASR) and average annual percentage change (AAPC) in incidence per 100,000 population of HIV and other sexually transmitted infections (STI) excluding HIV from 1990 to 2021 globally and in different geographic regions.

Characteristic	Incidence					DALYs				
	Case (*n*, 95% CI), 1990	ASR per 100,000 population (95% CI), 1990	Case (*n*, 95% CI), 2021	ASR per 100,000 population (95% CI), 2021	AAPC (95% CI), 1990–2021	Case (*n*, 95% CI), 1990	ASR per 100,000 population (95% CI), 1990	Case (*n*, 95% CI), 2021	ASR per 100,000 population (95% CI), 2021	AAPC (95% CI), 1990–2021
HIV										
Global	895,030 (802,918 to 993,220)	64.90 (64.90 to 64.90)	670,804 (596,594 to 762,161)	34.70 (34.70 to 34.70)	−1.94 (−2.21 to −1.67)	6,659,152 (4,755,670 to 9,617,289)	497.9 (497.80 to 497.90)	16,378,796 (14,479,032 to 18,796,594)	829.8 (829.70 to 829.80)	1.67 (1.20 to 2.14)
Region										
Andean Latin America	503 (286 to 1,392)	5 (5 to 5)	2049 (1,447 to 2,872)	11.50 (11.50 to 11.60)	2.91 (2.03 to 3.79)	4,830 (4,654 to 5,101)	55.10 (55 to 55.10)	32,512 (30,218 to 35,838)	184.4 (184.30 to 184.50)	4.19 (3.62 to 4.76)
Australasia	52 (40 to 65)	1 (1 to 1)	189 (88 to 321)	2.70 (2.70 to 2.70)	3.50 (2.43 to 4.57)	502 (468 to 546)	9.20 (9.20 to 9.20)	633 (501 to 884)	8.20 (8.20 to 8.20)	−0.55 (−3.52 to 2.50)
Caribbean	16,469 (11,504 to 22,861)	172.2 (172.10 to 172.30)	8,254 (4,353 to 13,478)	68.7 (68.60 to 68.70)	−2.92 (−3.10 to −2.75)	162,799 (106,982 to 257,110)	1773.90 (1773.60 to 1774.10)	146,185 (107,405 to 201,306)	1197.70 (1197.50 to 1197.90)	−1.17 (−2.48 to 0.15)
Central Asia	584 (408 to 838)	3.30 (3.30 to 3.30)	7,256 (4,962 to 11,301)	28.5 (28.50 to 28.60)	6.84 (4.98 to 8.74)	6,578 (6,510 to 6,659)	41.10 (41.10 to 41.20)	23,955 (23,121 to 25,144)	93.50 (93.50 to 93.60)	2.76 (1.94 to 3.57)
Central Europe	190 (124 to 268)	0.60 (0.60 to 0.60)	426 (261 to 655)	2 (2 to 2)	3.73 (2.95 to 4.52)	3,663 (3,586 to 3,752)	11.90 (11.90 to 11.90)	5,159 (4,808 to 5,719)	19.90 (19.80 to 19.90)	1.93 (0.65 to 3.22)
Central Latin America	3,410 (2,370 to 5,174)	8.10 (8.10 to 8.10)	6,992 (5,021 to 9,546)	10.30 (10.30 to 10.30)	0.75 (0.52 to 0.98)	43,158 (42,688 to 43,770)	111.80 (111.80 to 111.90)	122,352 (118,451 to 127,693)	177.80 (177.80 to 177.80)	1.51 (0.97 to 2.05)
Central Sub-Saharan Africa	68,971 (50,821 to 86,733)	553.20 (553.10 to 553.30)	48,790 (30,286 to 77,604)	152.50 (152.50 to 152.60)	−4.10 (−4.33 to −3.87)	548,900 (361,572 to 831,813)	4712.30 (4711.90 to 4712.70)	1,046,560 (786,739 to 1,415,228)	3593.10 (3592.90 to 3593.30)	−0.93 (−1.51 to −0.34)
East Asia	1876 (1,184 to 3,062)	0.60 (0.60 to 0.60)	4,693 (2,298 to 7,638)	1.60 (1.60 to 1.60)	3.39 (2.28 to 4.51)	17,269 (2,661 to 29,475)	5.50 (5.50 to 5.50)	202,974 (149,002 to 262,532)	54.90 (54.90 to 54.90)	7.70 (6.79 to 8.60)
Eastern Europe	1914 (1,482 to 2,629)	3.40 (3.40 to 3.40)	39,756 (30,731 to 53,933)	83.60 (83.60 to 83.60)	10.87 (10.10 to 11.63)	42,210 (41,588 to 43,292)	72.20 (72.20 to 72.30)	422,893 (406,926 to 443,996)	755.70 (755.60 to 755.80)	7.90 (7.15 to 8.66)
Eastern Sub-Saharan Africa	449,160 (385,001 to 519,612)	1013.20 (1013.10 to 1013.30)	189,069 (138,829 to 259,466)	173.90 (173.80 to 173.90)	−5.53 (−5.73 to −5.33)	3,759,617 (2,547,390 to 5,610,687)	9129.60 (9129.30 to 9129.90)	5,508,246 (4,768,438 to 6,444,676)	5,657 (5656.80 to 5657.10)	−1.53 (−2.08 to −0.97)
High-income Asia Pacific	168 (89 to 276)	0.40 (0.40 to 0.40)	336 (216 to 463)	1.10 (1.10 to 1.10)	3.41 (2.58 to 4.25)	445 (378 to 528)	1 (1 to 1)	1,146 (937 to 1,437)	2.8 (2.80 to 2.80)	3.48 (3.05 to 3.91)
High-income North America	13,083 (7,735 to 18,592)	17.50 (17.40 to 17.50)	15,613 (6,655 to 24,820)	18.60 (18.50 to 18.60)	0.74 (0.36 to 1.12)	164,336 (158,088 to 173,450)	206.40 (206.30 to 206.40)	62,269 (51,447 to 76,885)	68.90 (68.90 to 68.90)	−3.59 (−4.10 to −3.07)
North Africa and Middle East	1,416 (494 to 4,211)	1.80 (1.80 to 1.80)	11,234 (3,840 to 33,754)	7.10 (7 to 7.10)	4.49 (4.21 to 4.78)	12,204 (8,021 to 25,251)	16.80 (16.80 to 16.80)	225,249 (141,383 to 434,109)	139.30 (139.30 to 139.30)	7.02 (6.59 to 7.46)
Oceania	31 (17 to 52)	2 (2 to 2)	2043 (1,023 to 3,460)	58.80 (58.70 to 58.80)	11.71 (10.84 to 12.58)	408 (377 to 458)	28.30 (28.20 to 28.30)	20,671 (15,044 to 29,532)	619.30 (619.10 to 619.60)	10.44 (8.31 to 12.62)
South Asia	5,842 (3,459 to 9,315)	2.30 (2.30 to 2.30)	33,041 (20,041 to 60,804)	6.60 (6.60 to 6.60)	3.72 (2.56 to 4.89)	4,408 (2095 to 8,310)	1.80 (1.80 to 1.80)	970,048 (677,282 to 1,603,702)	200.70 (200.70 to 200.70)	16.58 (14.85 to 18.34)
Southeast Asia	16,297 (9,909 to 24,017)	12.80 (12.80 to 12.80)	24,819 (18,776 to 33,755)	13.80 (13.80 to 13.90)	−0.09 (−1.19 to 1.03)	153,232 (151,045 to 155,971)	127.50 (127.50 to 127.60)	406,855 (360,401 to 454,957)	217.30 (217.30 to 217.30)	2.16 (1.43 to 2.91)
Southern Latin America	1,408 (1,206 to 1,624)	11.30 (11.30 to 11.30)	2,599 (2,164 to 3,149)	14.90 (14.90 to 15)	0.87 (0.64 to 1.11)	7,828 (7,531 to 8,202)	64.10 (64.10 to 64.10)	29,139 (27,912 to 30,866)	160.30 (160.30 to 160.40)	3.33 (2.97 to 3.68)
Southern Sub-Saharan Africa	155,000 (135,675 to 173,316)	1120.70 (1120.50 to 1120.90)	121,228 (93,989 to 149,870)	552.10 (5,520 to 552.20)	−2.25 (−2.67 to −1.83)	617,060 (433,606 to 920,143)	4628.10 (4627.70 to 4628.40)	3,480,002 (3,183,802 to 3,795,428)	15941.30 (15940.80 to 15941.90)	3.92 (3.31 to 4.54)
Tropical Latin America	6,803 (5,280 to 8,789)	16.30 (16.30 to 16.30)	16,755 (10,195 to 25,618)	27.80 (27.80 to 27.80)	1.84 (1.28 to 2.4)	72,533 (70,722 to 74,963)	184.80 (184.70 to 184.80)	190,551 (180,823 to 203,432)	296.10 (296.10 to 296.20)	1.27 (0.51 to 2.05)
Western Europe	8,377 (7,180 to 9,933)	8.70 (8.70 to 8.80)	5,891 (4,337 to 7,403)	6.60 (6.60 to 6.60)	−0.94 (−1.26 to −0.61)	76,680 (74,752 to 79,065)	78.30 (78.30 to 78.40)	31,643 (27,821 to 36,310)	30.60 (30.60 to 30.60)	−3.17 (−4.39 to −1.94)
Western Sub-Saharan Africa	143,477 (117,682 to 170,879)	326.20 (326.10 to 326.20)	129,772 (105,130 to 157,634)	111 (111 to 111)	−3.46 (−3.62 to −3.29)	960,492 (626,484 to 1,515,188)	2330.50 (2330.40 to 2330.70)	3,449,756 (2,728,691 to 4,447,607)	3316.60 (3316.50 to 3316.70)	1.20 (0.41 to 2)
Other STIs										
Global	185,187,471 (154,089,641 to 225,253,879)	14050.40 (14050.30 to 14050.50)	286,514,651 (235,563,126 to 352,758,123)	14579.10 (14579.10 to 14579.20)	0.12 (0.09 to 0.14)	477,525 (310,553 to 759,127)	37.10 (37.10 to 37.10)	700,303 (432,099 to 1,135,938)	35.40 (35.40 to 35.50)	−0.15 (−0.18 to −0.11)
Region										
Andean Latin America	1,356,104 (1,125,249 to 1,616,339)	14707.20 (14706.40 to 14,708)	2,593,441 (2,144,899 to 3,149,604)	14692.90 (14692.40to 14693.50)	0 (−0.08 to 0.08)	3,772 (2,223 to 6,213)	42.80 (42.80 to 42.90)	7,228 (4,117 to 12,238)	41.10 (41 to 41.10)	−0.13 (−0.19 to −0.08)
Australasia	360,445 (292,653 to 447,258)	6632.90 (6632.20 to 6633.50)	490,881 (393,440 to 609,515)	6547.10 (6546.50 to 6547.70)	−0.03 (−0.12 to 0.05)	1,695 (991 to 2,752)	30.80 (30.70 to 30.80)	2,195 (1,277 to 3,630)	28.70 (28.60 to 28.70)	−0.23 (−0.29 to −0.17)
Caribbean	2,029,631 (1,690,115 to 2,466,543)	22,246 (22,245 to 22,247)	2,699,532 (2,212,468 to 3,298,612)	22258.30 (22257.40 to 22259.10)	0 (0 to 0.01)	4,356 (2,855 to 6,916)	49.70 (49.60 to 49.70)	6,603 (4,340 to 10,249)	54.30 (54.20 to 54.30)	0.30 (0.09 to 0.52)
Central Asia	4,345,015 (3,482,806 to 5,323,118)	25554.10 (25553.30 to 25554.90)	6,201,279 (5,075,624 to 7,620,481)	25,030 (25029.40 to 25030.60)	−0.07 (−0.09 to −0.06)	6,622 (4,363 to 10,018)	41.80 (41.70 to 41.80)	8,584 (4,925 to 14,319)	33.90 (33.90 to 33.90)	−0.70 (−0.86 to −0.53)
Central Europe	4,507,473 (3,744,071 to 5,454,278)	14,619 (14618.60 to 14619.50)	3,746,765 (3,093,149 to 4,608,360)	14380.70 (14380.30 to 14381.20)	−0.05 (−0.07 to −0.03)	7,361 (4,336 to 12,102)	23.10 (23.10 to 23.10)	6,143 (3,411 to 10,588)	21.60 (21.60 to 21.60)	−0.21 (−0.26 to −0.16)
Central Latin America	10,330,727 (8,437,832 to 12,596,668)	26049.10 (26048.50 to 26049.60)	17,623,488 (14,323,297 to 21,934,513)	25673.40 (25673.10 to 25673.80)	−0.06 (−0.14 to 0.01)	19,084 (10,879 to 33,409)	49.90 (49.90 to 50)	33,805 (18,901 to 59,158)	49.10 (49.10 to 49.10)	−0.05 (−0.07 to −0.02)
Central Sub-Saharan Africa	1,986,031 (1,682,416 to 2,329,021)	16272.80 (16,272 to 16273.50)	5,168,735 (4,333,793 to 6,134,557)	16113.20 (16112.70 to 16113.60)	−0.04 (−0.06 to −0.02)	9,428 (6,158 to 14,508)	83 (82.90 to 83)	19,764 (12,171 to 32,134)	65.40 (65.30 to 65.40)	−0.79 (−0.91 to −0.66)
East Asia	46,678,760 (38,745,529 to 57,796,724)	14426.50 (14426.40 to 14426.60)	50,834,670 (41,195,911 to 65,454,902)	14416.30 (14416.20 to 14416.50)	−0.01 (−0.12 to 0.11)	68,597 (39,643 to 118,850)	22.40 (22.40 to 22.40)	76,166 (41,489 to 134,300)	20.30 (20.30 to 20.30)	−0.33 (−0.39 to −0.27)
Eastern Europe	9,342,510 (7,699,225 to 11,630,235)	16438.20 (16437.90 to 16438.60)	7,939,829 (6,562,318 to 10,047,353)	15984.40 (15,984 to 15984.7)	−0.09 (−0.12 to −0.06)	18,587 (12,356 to 28,404)	32 (31.90 to 32)	16,746 (10,980 to 25,879)	29.90 (29.90 to 29.90)	−0.23 (−0.46 to 0.01)
Eastern Sub-Saharan Africa	11,267,568 (9,356,703 to 13,387,302)	26,975 (26974.50 to 26975.50)	26,978,756 (22,156,148 to 32,433,050)	26203.70 (26203.40 to 26,204)	−0.10 (−0.12 to −0.08)	53,449 (34,227 to 81,735)	125.30 (125.30 to 125.30)	89,208 (55,495 to 140,121)	85.10 (85 to 85.10)	−1.25 (−1.29 to −1.21)
High-income Asia Pacific	3,507,767 (2,820,700 to 4,447,972)	7627.10 (7626.90 to 7627.40)	2,955,225 (2,357,306 to 3,870,508)	7359.80 (7359.50 to 7360.10)	−0.11 (−0.14 to −0.07)	13,770 (7,714 to 23,292)	29.50 (29.50 to 29.50)	12,275 (6,963 to 20,708)	29.50 (29.50 to 29.50)	0.01 (−0.03 to 0.05)
High-income North America	9,031,095 (6,789,233 to 12,008,130)	11603.30 (11,603 to 11603.50)	9,965,323 (7,518,830 to 13,451,526)	11,414 (11413.80 to 11414.20)	−0.06 (−0.21 to 0.10)	26,273 (14,224 to 45,904)	33.50 (33.40 to 33.50)	27,552 (14,930 to 48,943)	31.30 (31.30 to 31.30)	−0.22 (−0.25 to −0.19)
North Africa and Middle East	11,485,541 (9,524,893 to 13,616,376)	14757.40 (14757.10 to 14757.70)	21,452,038 (17,779,361 to 26,025,628)	13408.60 (13408.40 to 13408.80)	−0.30 (−0.41 to −0.20)	15,482 (8,461 to 27,128)	21.80 (21.80 to 21.80)	32,866 (17,390 to 58,418)	20.30 (20.30 to 20.40)	−0.21 (−0.38 to −0.05)
Oceania	650,397 (545,757 to 767,858)	41913.30 (41,910 to 41916.50)	1,388,458 (1,172,884 to 1,655,967)	39824.70 (39822.60 to 39826.80)	−0.16 (−0.28 to −0.03)	903 (508 to 1,571)	64.30 (64.20 to 64.40)	1900 (998 to 3,520)	56.20 (56.10 to 56.30)	−0.43 (−0.60 to −0.26)
South Asia	21,189,375 (17,541,356 to 25,992,289)	8603.10 (8602.90 to 8603.20)	42,373,342 (34,783,920 to 52,433,099)	8,617 (8616.9 to 8617.10)	0 (−0.07 to 0.08)	121,477 (86,275 to 165,818)	50.70 (50.70 to 50.70)	174,156 (116,461 to 255,497)	35.90 (35.90 to 35.90)	−1.12 (−1.32 to −0.92)
Southeast Asia	16,796,661 (13,760,237 to 20,401,141)	14556.60 (14556.40 to 14556.80)	26,052,565 (21,112,448 to 32,526,560)	14002.90 (14002.70 to 14003.10)	−0.09 (−0.25 to 0.06)	25,747 (13,952 to 45,967)	23.10 (23.10 to 23.10)	41,180 (22,535 to 73,778)	22 (22 to 22)	−0.15 (−0.17 to −0.13)
Southern Latin America	960,046 (804,559 to 1,154,024)	7788.70 (7788.30 to 7789.20)	1,408,502 (1,168,525 to 1,728,448)	7966.20 (7965.80 to 7966.60)	0.08 (0.01 to 0.14)	4,194 (2,466 to 6,854)	34.40 (34.40 to 34.50)	5,899 (3,436 to 9,789)	32.80 (32.70 to 32.80)	−0.16 (−0.23 to −0.10)
Southern Sub-Saharan Africa	5,973,052 (5,016,619 to 7,061,955)	45,851 (45849.80 to 45852.20)	9,088,183 (7,587,114 to 11,073,463)	41462.60 (41461.80 to 41463.50)	−0.32 (−0.35 to −0.29)	15,072 (94,370 to 24,307)	121.50 (121.40 to 121.50)	18,501 (10,376 to 32,109)	84.50 (84.50 to 84.60)	−1.13 (−1.40 to −0.85)
Tropical Latin America	9,533,164 (7,805,731 to 11,760,736)	24526.40 (24525.90 to 24526.90)	15,369,432 (12,574,277 to 19,243,800)	24,585 (24584.60 to 24585.40)	0 (−0.06 to 0.06)	15,716 (9,295 to 26,808)	41.60 (41.60 to 41.60)	31,897 (18,579 to 53,848)	50.20 (50.20 to 50.30)	0.63 (0.53 to 0.74)
Western Europe	3,700,145 (3,027,386 to 4,603,532)	3805.10 (3804.90 to 3805.20)	3,626,190 (2,945,629 to 4,617,424)	3764.70 (3764.60 to 3764.80)	−0.02 (−0.08 to 0.03)	12,791 (7,130 to 21,650)	13 (13 to 13)	14,213 (7,956 to 24,180)	14.10 (14.10 to 14.10)	0.25 (0.19 to 0.32)
Western Sub-Saharan Africa	10,155,964 (8,440,007 to 12,214,588)	24355.80 (24355.30 to 24356.30)	28,558,016 (23,352,692 to 34,624,980)	25063.10 (25062.80 to 25063.40)	0.09 (0.06 to 0.11)	33,149 (20,001 to 53,586)	82.40 (82.40 to 82.40)	73,421 (42,039 to 123,883)	66.30 (66.30 to 66.30)	−0.71 (−0.79 to −0.63)

The global burden of five common STIs among women of reproductive age reached 286,514,651 (95% CI: 23,556, 3,126–35,275, 8,123) new cases in 2021, with an age-standardized incidence rate of 14,579.10 cases per 100,000 population (95% CI: 14,579.10–14,579.20) (Table 1). The incidence rate of STIs exhibited a gradual annual increase of 0.12% (95% CI: 0.09–0.14) from 1990 to 2021. In the same year, STI-related DALYs totaled 700,303 (95% CI: 432,099–1,135,938), with an age-standardized DALY rate of 35.4 per 100,000 population (95% CI: 35.40–35.50). The burden of STIs, as measured by DALYs, showed a slight annual decline of −0.15% (95% CI: −0.18 to −0.11) globally over the study period. As detailed in Table 1.

**Figure 1:**
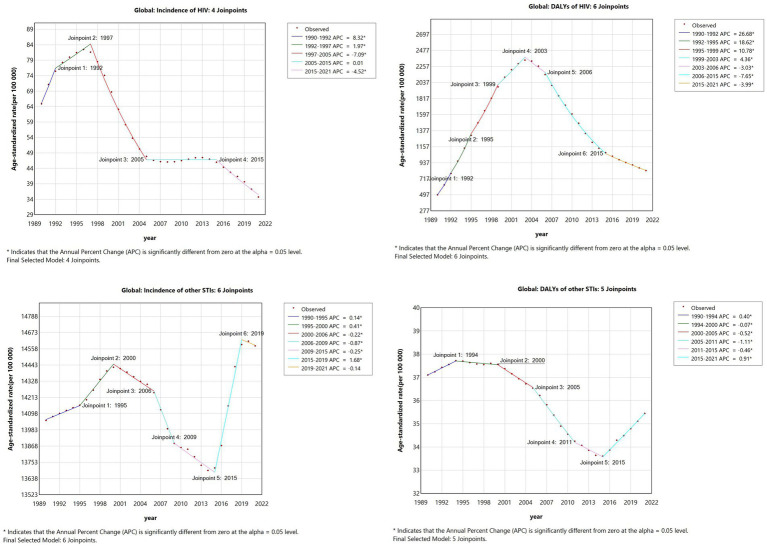
Joinpoint regression analysis of global HIV incidence (Panel **A**) and DALYs (Panel **B**) and joinpoint regression analysis of global other STIs incidence (Panel **C**) and DALYs (Panel **D**) among women of childbearing aged 15–49 years from 1990 to 2021. The joinpoints have been labeled in the figure, and the corresponding APC is shown in the legend. Different colors represent different segments. All values are per 100,000 population. APC, annual percentage change; DALYs, disability-adjusted life-years.

Joinpoint regression analysis revealed distinct temporal trends in HIV incidence among women of reproductive age. Between 1990 and 1997, the age-standardized incidence rate increased, followed by a sharp decline from 1997 to 2005, with the steepest rate of reduction observed during this period (Figure 1). From 2005 to 2015, incidence rates stabilized, with a subsequent gradual decline from 2015 to 2021. In contrast, the DALY rate exhibited a fluctuating pattern, increasing steadily from 1990 to 2003 before peaking in 2003. Thereafter, a consistent downward trend in HIV-related DALYs was observed from 2003 to 2021.

Between 1990 and 2021, trends in HIV incidence exhibited greater homogeneity compared to other STIs, which demonstrated a more complex trajectory. The age-standardized incidence rates of STIs increased from 1990 to 2000, followed by a sustained decline between 2000 and 2015. A sharp rise occurred from 2015 to 2019, peaking in 2019, before declining again from 2019 to 2021. In terms of STI-related DALYs, a gradual increase was observed from 1990 to 1994, followed by a prolonged high plateau between 1994 and 2000. A steady decline occurred from 2000 to 2015, but a renewed increase was noted between 2015 and 2021. The overall trend in STI-related DALYs displayed a fluctuating pattern, with a notable rise from 1990 to 2003, peaking in 2003, before exhibiting a consistent downward trajectory from 2003 to 2021. As shown in Figure 1.

### Analysis by geographical regions

**Figure 2:**
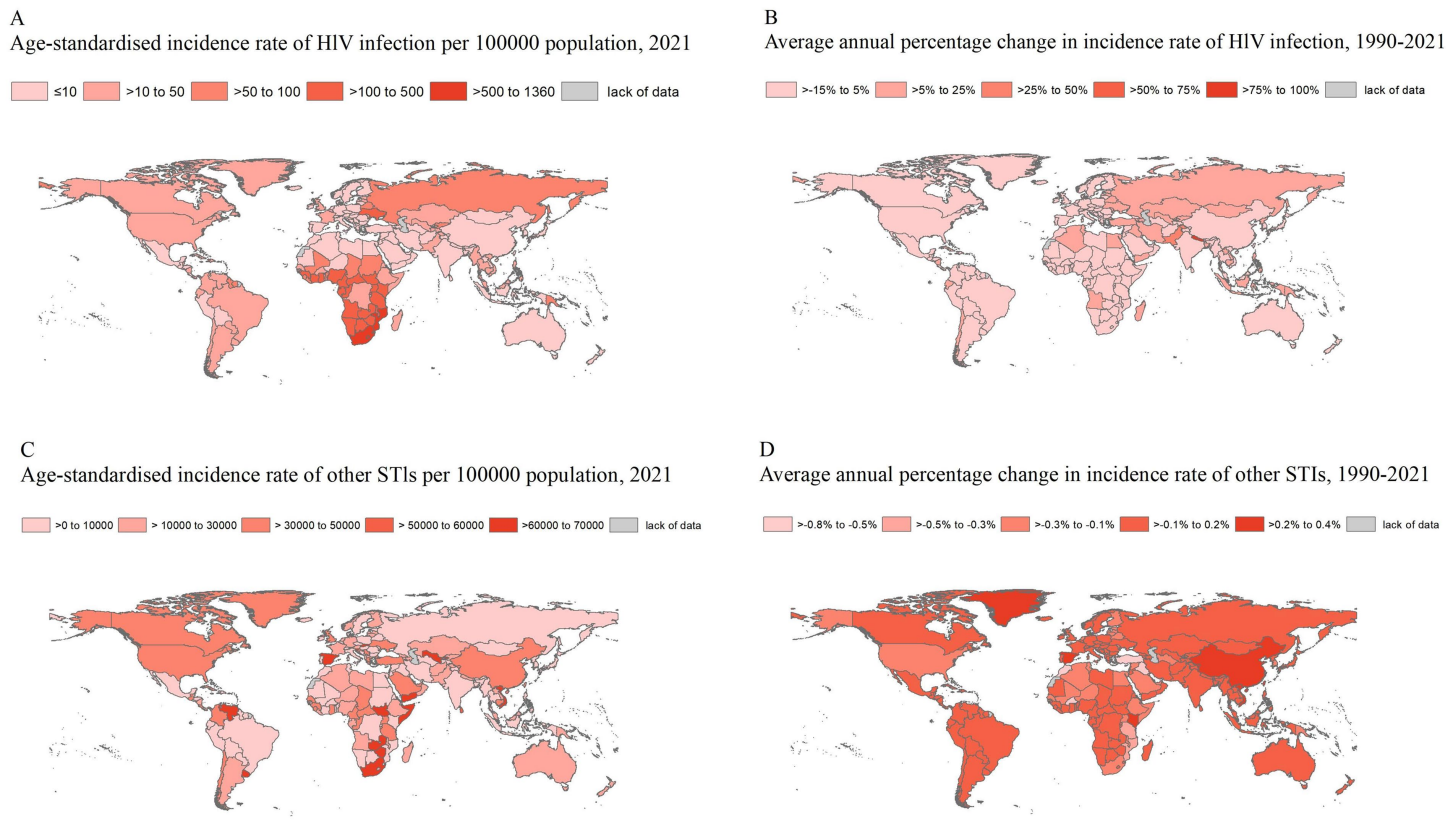
Age-standardized incidence rates in 2021 and average annual percentage changes in incidence rates from 1990 to 2021 for HIV and other STIs, by country. Age-standardized rate **(A)** and annual percentage change in rate **(B)** of the incidence of HIV. Age-standardized rate **(C)** and annual percentage change in rate **(D)** of the incidence of other STIs.

In 2021, southern sub-Saharan Africa recorded the highest age-standardized incidence and DALY rates of HIV (Table 1; Figure 2). Between 1990 and 2021, the most pronounced increase in HIV incidence occurred in Oceania (AAPC: 11.71, 95% CI: 10.84–12.58) and Eastern Europe (AAPC: 10.87, 95% CI: 10.1–11.63) (Table 1). Over the same period, the greatest increase in HIV-related DALYs was observed in South Asia (AAPC: 16.58, 95% CI: 14.85–18.34) and Oceania (AAPC: 10.44, 95% CI: 8.31–12.62).

In 2021, the highest age-standardized incidence rates of STIs were reported in southern sub-Saharan Africa, Oceania, and western sub-Saharan Africa (Table 1; Figure 2). Notably, the highest HIV incidence and DALY rates were predominantly concentrated in southern and western sub-Saharan Africa. The AAPC of STI-related DALYs remained stable or exhibited a downward trend in most regions. However, from 1990 to 2021, STI-related DALYs showed an increasing trend in Western Europe, Tropical Latin America, the Caribbean, and the High-income Asia-Pacific region, whereas all other GBD regions experienced a decline.

### Analysis by SDI regions

An apparent trend emerged across SDI regions (Table 2), where higher SDI levels were generally associated with lower HIV incidence. Conversely, in lower SDI regions, the decline in STI-related DALY rates was more pronounced.

**Table 2:** Number of cases, age-standardized rate (ASR) and average annual percentage change (AAPC) in incidence per 100,000 population of HIV and other sexually transmitted infections (STI) excluding HIV from 1990 to 2021 in different sociodemographic Index regions (SDI).

Characteristic	Incidence					DALYs				
	Case (*n*, 95% CI), 1990	ASR per 100,000 population (95% CI), 1990	Case (*n*, 95% CI), 2021	ASR per 100,000 population (95% CI), 2021	AAPC (95% CI), 1990–2021	Case (*n*, 95% CI), 1990	ASR per 100,000 population (95% CI), 1990	Case (*n*, 95% CI), 2021	ASR per 100,000 population (95% CI), 2021	AAPC (95% CI), 1990–2021
HIV										
High SDI	18,447 (12,518 to 24,795)	8.10 (8.10 to 8.10)	21,790 (11,605 to 31,997)	9.10 (9.10 to 9.10)	0.47 (−0.46 to 1.41)	212,181 (205,046 to 222,375)	89.60 (89.60 to 89.70)	111,855 (97,442 to 130,551)	41.60 (41.60 to 41.60)	−2.54 (−3.37 to −1.71)
High-middle SDI	9,498 (8,317 to 10,892)	3.40 (3.40 to 3.40)	50,530 (39,303 to 67,253)	16.50 (16.50 to 16.50)	5.14 (4.61 to 5.68)	104,061 (101,330 to 106,840)	37.30 (37.30 to 37.30)	563,186 (525,281 to 623,920)	164.80 (164.80 to 164.80)	4.91 (4.28 to 5.55)
Middle SDI	77,507 (66,288 to 88,596)	16.70 (16.70 to 16.70)	185,428 (158,205 to 212,803)	30.70 (30.70 to 30.70)	1.92 (1.32 to 2.53)	411,167 (374,639 to 458,254)	93.80 (93.80 to 93.80)	4,258,659 (3,953,295 to 4,583,796)	666.70 (666.70 to 666.70)	6.47 (5.37 to 7.59)
Low-middle SDI	347,713 (314,713 to 384,567)	122.90 (122.80 to 122.90)	201,844 (168,488 to 247,814)	39.50 (39.40 to 39.50)	−3.59 (−3.78 to −3.41)	1,626,302 (1,116,229 to 2,518,026)	596.30 (596.20 to 596.30)	5,523,174 (4,535,235 to 6,835,621)	1119.80 (1119.80 to 1119.80)	2.12 (1.58 to 2.66)
Low SDI	440,945 (367,285 to 520,786)	385.10 (385 to 385.10)	210,619 (157,932 to 276,386)	76.10 (76.10 to 76.10)	−5.12 (−5.33 to −4.92)	4,296,830 (2,911,625 to 6,344,602)	3993.60 (3993.50 to 3993.70)	5,910,328 (5,132,769 to 6,964,977)	2351.70 (2351.60 to 2351.70)	−1.73 (−2.57 to −0.88)
Other STIs										
High SDI	21,237,353 (17,025,840 to 26,636,631)	9127.80 (9127.60 to 9127.90)	23,877,905 (19,042,009 to 30,333,545)	9357.70 (9357.60 to 9357.80)	0.11 (0 to 0.22)	59,249 (32,592 to 101,286)	25.10 (25.10 to 25.10)	63,535 (35,310 to 109,019)	24.20 (24.20 to 24.20)	−0.12 (−0.14 to −0.1)
High-middle SDI	36,285,597 (30,273,388 to 44,717,451)	13027.80 (13027.70 to 13027.90)	40,956,889 (33,315,364 to 51,538,860)	12851.30 (12851.20 to 12851.40)	−0.04 (−0.1 to 0.02)	63,561 (38,009 to 105,709)	23.40 (23.40 to 23.40)	75,873 (43,547 to 127,965)	22.30 (22.30 to 22.30)	−0.12 (−0.21 to −0.03)
Middle SDI	71,331,023 (59,385,710 to 86,936,841)	16545.90 (16545.80 to 16,546)	101,099,248 (82,826,898 to 126,223,497)	15970.60 (15970.50 to 15970.70)	−0.11 (−0.15 to −0.08)	132,586 (81,913 to 219,713)	32.20 (32.20 to 32.20)	196,583 (114,149 to 333,249)	30.50 (30.50 to 30.50)	−0.18 (−0.22 to −0.13)
Low-middle SDI	35,212,603 (29,280,344 to 42,370,305)	13327.80 (13327.60 to 13327.90)	69,405,892 (56,991,667 to 84,676,741)	13815.90 (13815.80 to 13,816)	0.11 (0.05 to 0.16)	126,706 (87,047 to 185,524)	49.80 (49.80 to 49.80)	200,617 (128,342 to 313,234)	40.50 (40.50 to 40.50)	−0.68 (−0.75 to −0.6)
Low SDI	20,896,331 (17,461,251 to 24,909,446)	19187.50 (19187.20 to 19187.70)	50,882,895 (42,215,953 to 61,000,438)	19207.90 (19207.80 to 19208.10)	−0.01 (−0.05 to 0.03)	95,003 (63,096 to 141,796)	88.30 (88.30 to 88.40)	163,116 (104,410 to 256,626)	62.50 (62.50 to 62.50)	−1.13 (−1.24 to −1.02)

### Analysis by age group

In 2021, the highest age-standardized incidence rate of HIV was observed in the 25–29 years age group (Table 3). Across all seven age groups, HIV incidence exhibited a downward trend, with the most significant reduction occurring in individuals aged 45–49 years (AAPC: -2.70, 95% CI: −2.88 to −2.51) between 1990 and 2021. The highest age-standardized DALY rate of HIV in 2021 was recorded in the 30–34 years age group. Over the same period, HIV-related DALY rates increased across all age groups, with the most substantial rise occurring in individuals aged 40–44 years (AAPC: 3.25, 95% CI: 2.62 to 3.88). In contrast, STI incidence trends varied by age. While the 15–19 years age group exhibited a decline in STI incidence, all other age groups showed an upward trajectory. The highest age-standardized incidence rate of STIs in 2021 was recorded in the 30–34 years age group, whereas the highest STI-related DALY rate occurred in the 40–44 years age group.

**Table 3:** Number of cases, age-standardized rate (ASR) and average annual percentage change (AAPC) in incidence, prevalence and DALYs per 100,000 population of HIV and other sexually transmitted infections (STI) excluding HIV from 1990 to 2021 in different age groups.

Characteristic	Age	Case (*n*, 95% CI), 1990	ASR per 100,000 population (95% CI), 1990	Case (*n*, 95% CI), 2021	ASR per 100,000 population (95% CI), 2021	AAPC (95% CI), 1990–2021
HIV						
Incidence	15–19 years	186,689 (169,021 to 206,759)	73.10 (66.10 to 80.90)	101,535 (87,962 to 123,663)	33.40 (29 to 40.70)	−2.51 (−2.76 to −2.25)
	20–24 years	179,695 (157,340 to 201,659)	73.60 (64.40 to 82.60)	107,382 (92,511 to 125,926)	36.60 (31.50 to 42.90)	−2.17 (−2.47 to −1.87)
	25–29 years	218,954 (194,270 to 246,560)	99.50 (88.30 to 112)	171,966 (150,215 to 198,323)	59.10 (51.60 to 68.20)	−1.67 (−1.82 to −1.53)
	30–34 years	117,442 (102,121 to 133,828)	61.80 (53.70 to 70.40)	112,529 (99,913 to 127,177)	37.60 (33.40 to 42.50)	−1.58 (−1.78 to −1.38)
	35–39 years	88,038 (76,106 to 101,357)	50.80 (43.90 to 58.40)	82,146 (69,057 to 95,866)	29.60 (24.90 to 34.50)	−1.74 (−2.03 to −1.44)
	40–44 years	59,624 (52,110 to 69,758)	42.50 (37.20 to 49.70)	55,879 (47,573 to 64,796)	22.50 (19.20 to 26.10)	−1.99 (−2.48 to −1.50)
	45–49 years	44,588 (38,254 to 51,523)	39.20 (33.60 to 45.30)	39,367 (32,534 to 46,735)	16.70 (13.80 to 19.80)	−2.70 (−2.88 to −2.51)
Prevalence	15–19 years	451,251 (410,633 to 497,687)	176.60 (160.70 to 194.80)	674,377 (616,162 to 761,637)	222.10 (202.90 to 250.80)	0.76 (0.53 to 0.99)
	20–24 years	775,488 (713,027 to 841,648)	317.60 (292.10 to 344.70)	1,411,907 (1,299,137 to 1,554,886)	480.60 (442.30 to 529.30)	1.31 (1.07 to 1.56)
	25–29 years	873,672 (803,861 to 941,788)	396.90 (365.20 to 427.90)	2,422,267 (2,271,291 to 2,621,332)	832.40 (780.50 to 900.80)	2.43 (2.23 to 2.62)
	30–34 years	613,504 (557,501 to 666,884)	322.70 (293.30 to 350.80)	3,160,991 (2,991,857 to 3,361,226)	1057.40 (1000.90 to 1124.40)	3.89 (3.73 to 4.04)
	35–39 years	394,355 (357,567 to 431,074)	227.40 (206.10 to 248.50)	3,363,638 (3,221,400 to 3,519,801)	1210.80 (1159.60 to 1,267)	5.54 (5.32 to 5.77)
	40–44 years	263,154 (239,331 to 287,174)	187.70 (170.70 to 204.80)	3,016,072 (2,865,467 to 3,165,378)	1215.70 (1,155 to 1275.9)	6.21 (5.98 to 6.45)
	45–49 years	183,409 (167,420 to 203,811)	161.20 (147.10 to 179.10)	2,448,621 (2,319,532 to 2,560,735)	1039.10 (984.30 to 1086.70)	6.19 (6.07 to 6.31)
DALYs	15–19 years	529,950 (373,362 to 817,021)	207.40 (146.10 to 319.70)	981,251 (721,531 to 1,302,674)	323.20 (237.60 to 429)	1.36 (1.07 to 1.65)
	20–24 years	1,263,353 (884,032 to 1,887,789)	517.50 (362.10 to 773.30)	1,771,823 (1,388,055 to 2,276,614)	603.20 (472.50 to 775)	0.42 (0 to 0.85)
	25–29 years	1,667,222 (1,028,754 to 2,483,458)	757.50 (467.40 to 1128.30)	2,639,091 (2,054,920 to 3,468,207)	906.90 (706.20 to 1191.90)	0.49 (−0.03 to 1.02)
	30–34 years	1,337,195 (876,828 to 1,956,385)	703.40 (461.20 to 1029.10)	3,322,270 (2,635,847 to 4,227,834)	1111.40 (881.80 to 1414.30)	1.43 (1.03 to 1.83)
	35–39 years	854,096 (529,046 to 1,297,471)	492.40 (305 to 748)	3,082,365 (2,511,640 to 3,866,311)	1109.60 (904.10 to 1391.70)	2.68 (2.20 to 3.16)
	40–44 years	562,035 (358,704 to 836,644)	400.80 (255.80 to 596.60)	2,629,324 (2,148,601 to 3,217,775)	1059.80 (866.10 to 1,297)	3.25 (2.62 to 3.88)
	45–49 years	445,301 (282,155 to 651,300)	391.30 (247.90 to 572.30)	1,952,673 (1,541,993 to 2,638,580)	828.70 (654.40 to 1119.70)	2.46 (2.12 to 2.81)
Other STIs						
Incidence	15–19 years	16,050,222 (12,371,752 to 20,441,528)	6280.90 (4841.40 to 7999.40)	19,000,256 (14,799,961 to 23,620,918)	6257.30 (4,874 to 7,779)	−0.01 (−0.08 to 0.05)
	20–24 years	31,998,676 (23,939,338 to 39,979,463)	13,107 (9805.80 to 16,376)	38,949,578 (28,932,905 to 48,486,799)	13259.30 (9849.40 to 16,506)	0.04 (0 to 0.08)
	25–29 years	36,712,718 (27,450,042 to 47,754,538)	16,680 (12471.60 to 21696.80)	50,444,651 (37,775,090 to 65,884,624)	17335.70 (12981.70 to 22641.80)	0.13 (0.09 to 0.17)
	30–34 years	34,572,935 (23,946,414 to 47,743,392)	18185.80 (12596.10 to 25113.70)	56,965,937 (39,176,713 to 78,716,761)	19056.60 (13105.60 to 26332.80)	0.16 (0.10 to 0.23)
	35–39 years	30,556,027 (21,441,122 to 40,055,461)	17616.30 (12361.30 to 23092.90)	51,190,065 (35,874,316 to 67,580,768)	18426.80 (12913.60 to 24326.90)	0.14 (0.09 to 0.19)
	40–44 years	21,697,631 (14,868,661 to 31,499,120)	15473.40 (10603.40 to 22463.20)	40,526,414 (27,619,419 to 59,268,265)	16335.40 (11132.80 to 23889.80)	0.18 (0.11 to 0.24)
	45–49 years	13,599,261 (9,208,848 to 21,474,119)	11950.10 (8092.10 to 18869.90)	29,437,751 (19,788,186 to 46,722,312)	12492.50 (8397.50 to 19827.60)	0.14 (0.09 to 0.19)
Prevalence	15–19 years	20,740,426 (16,858,995 to 24,998,988)	8116.30 (6597.40 to 9782.90)	26,143,411 (20,991,640 to 31,844,375)	8609.70 (6913.10 to 10487.20)	0.19 (0.15 to 0.23)
	20–24 years	44,754,239 (37,375,008 to 52,786,878)	18331.80 (15309.20 to 21,622)	57,005,172 (47,277,440 to 68,488,175)	19405.90 (16094.30 to 23,315)	0.19 (0.11 to 0.27)
	25–29 years	59,137,842 (50,117,983 to 68,986,369)	26868.70 (22770.6 to 31343.3)	81,673,726 (68,925,352 to 95,720,855)	28067.80 (23686.7 to 32895.2)	0.15 (0.11 to 0.2)
	30–34 years	62,057,259 (53,113,578 to 73,401,303)	32,643 (27938.50 to 38610.10)	100,045,631 (84,936,189 to 118,914,615)	33467.80 (28413.30 to 39,780)	0.08 (0.04 to 0.11)
	35–39 years	61,107,615 (52,909,270 to 69,877,603)	35,230 (30503.50 to 40286.10)	101,418,460 (87,004,265 to 116,647,013)	36507.40 (31318.70 to 41989.20)	0.11 (0.07 to 0.15)
	40–44 years	51,068,857 (44,418,616 to 58,479,506)	36419.10 (31676.60 to 41703.90)	93,693,634 (81,101,112 to 107,819,672)	37,766 (32690.20 to 43459.90)	0.12 (0.08 to 0.15)
	45–49 years	40,021,793 (34,561,154 to 45,461,780)	35168.30 (30369.90 to 39948.60)	84,474,206 (72,560,615 to 96,583,857)	35848.40 (30792.70 to 40987.40)	0.07 (0.03 to 0.1)
DALYs	15–19 years	32,656 (23,448 to 45,988)	12.80 (9.20 to 18)	37,444 (26,207 to 54,994)	12.30 (8.60 to 18.1)	−0.11 (−0.26 to 0.03)
	20–24 years	60,768 (41,159 to 93,584)	24.90 (16.90 to 38.30)	73,552 (46,722 to 120,365)	25 (15.90 to 41)	0.03 (−0.05 to 0.11)
	25–29 years	88,011 (56,388 to 136,771)	40 (25.60 to 62.10)	112,167 (67,259 to 178,180)	38.50 (23.10 to 61.20)	−0.10 (−0.18 to −0.03)
	30–34 years	89,595 (55,865 to 140,796)	47.10 (29.40 to 74.10)	133,128 (77,571 to 216,014)	44.50 (25.90 to 72.30)	−0.18 (−0.27 to −0.10)
	35–39 years	85,927 (52,641 to 142,357)	49.50 (30.30 to 82.10)	134,169 (78,940 to 228,785)	48.30 (28.40 to 82.40)	−0.07 (−0.13 to −0.01)
	40–44 years	75,436 (45,265 to 130,492)	53.80 (32.30 to 93.10)	126,169 (72,595 to 223,929)	50.90 (29.30 to 90.30)	−0.18 (−0.24 to −0.12)
	45–49 years	45,132 (27,943 to 76,142)	39.70 (24.60 to 66.90)	83,673 (46,808 to 148,624)	35.50 (19.90 to 63.10)	−0.35 (−0.42 to −0.28)

**Figure 3:**
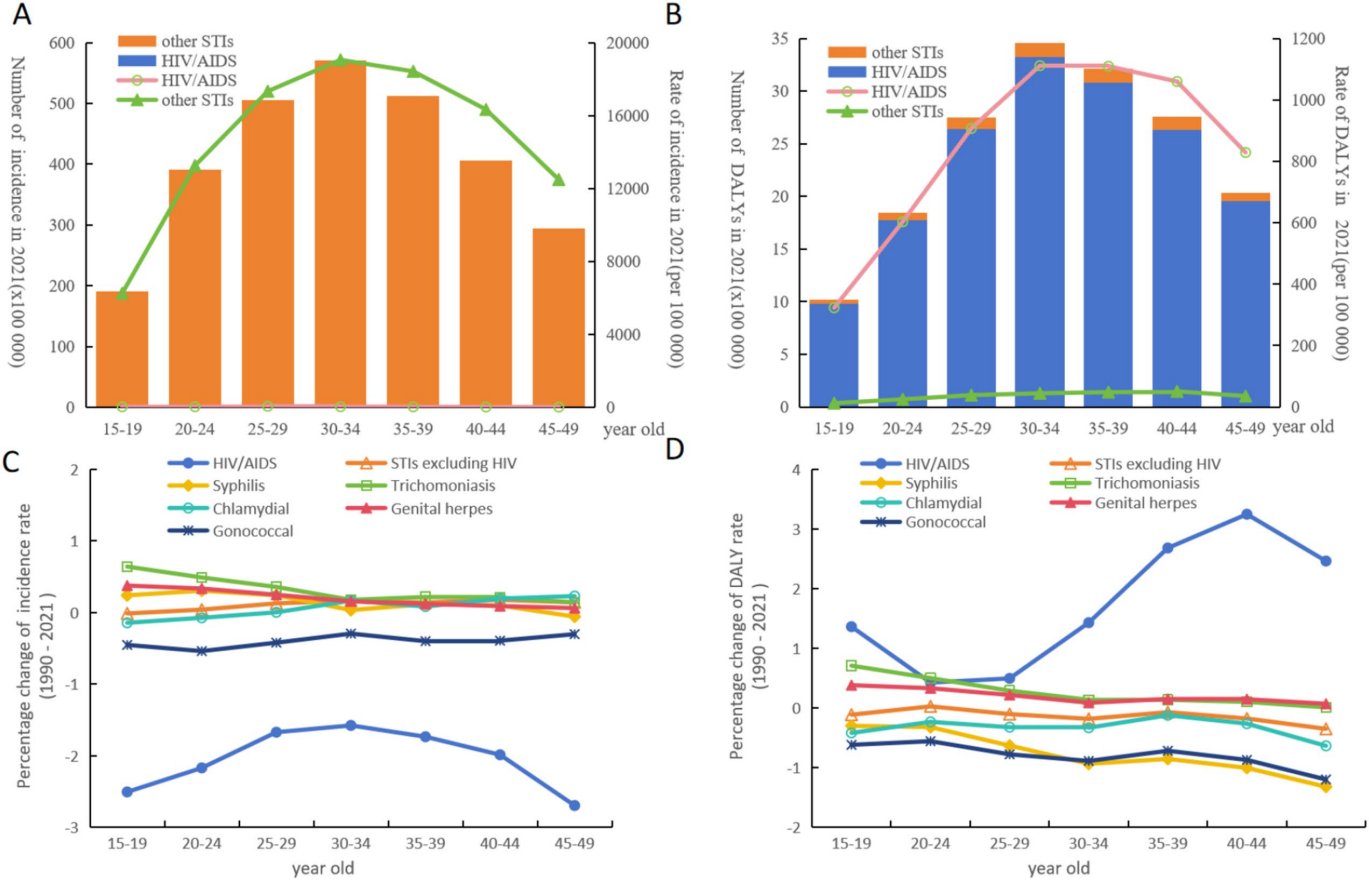
Numbers and rates of incident **(A)** and DALYs **(B)** of HIV and other STIs in different age brackets. Percentage changes of incidence rate **(C)** and DALY rate **(D)** of HIV and other STIs in different age brackets.

Figure 3 illustrates the distribution of HIV and STI incidence and DALY rates across different age groups, with both bar and line graphs displaying a characteristic mountain-shaped trend—peaking in middle-aged groups (30–34 years) and declining at both younger (15–19 years) and older (45–49 years) age groups.

### Analysis by specific STIs and HIV

**Figure 4:**
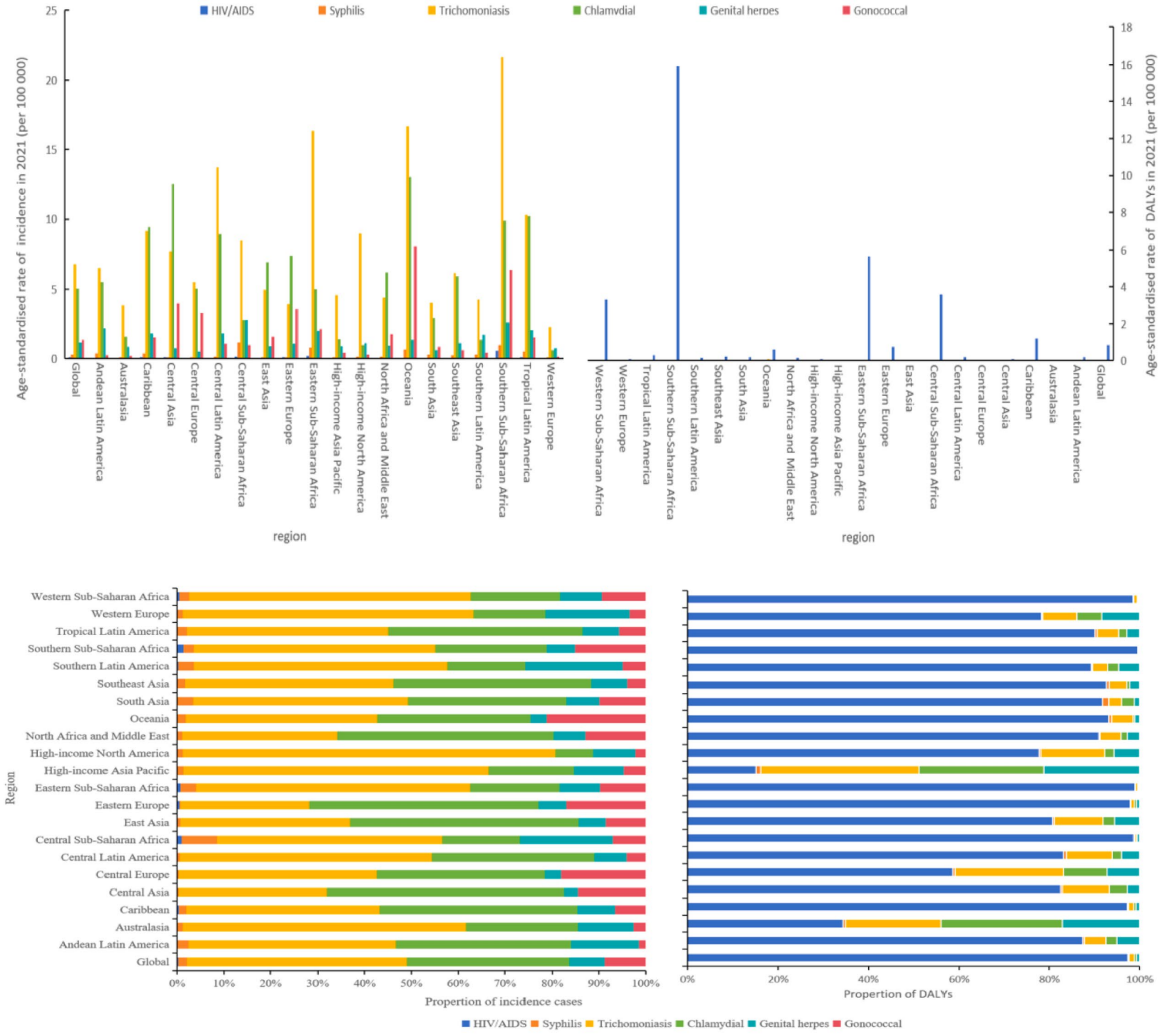
Age-standardized incidence and DALY rates of HIV, syphilis, trichmoniasis, chlamydia, genital herpes and gonorrhea, and proportions of incident cases and DALYs contributed by each infection, globally and for 21 GBD regions, 2021.

Among specific STIs, incidence patterns varied geographically. With the exception of gonococcal infection, which exhibited a slight decline, other STIs showed increasing incidence trends. In 2021, the highest incidence rates were observed in different regions: syphilis in Central Sub-Saharan Africa (1,142.40 per 100,000 population), chlamydial infection in Oceania (13,034.80), gonococcal infection in Oceania (8,089.20), trichomoniasis in Southern Sub-Saharan Africa (21,664.70), and genital herpes in Central Sub-Saharan Africa (2,783) (Figure 4; Supplementary Tables S1–S5).

Oceania, Eastern Sub-Saharan Africa, and Central Latin America were identified as high-incidence regions for trichomoniasis. In 2021, trichomoniasis accounted for the largest proportion of STI cases globally. Chlamydia was the second most prevalent STI, with particularly high incidence rates in Oceania, Central Asia, and Tropical Latin America. As depicted in Figure 4, trichomoniasis and chlamydia comprised the majority of STI cases worldwide.

### Analysis by ARIMA

**Figure 5:**
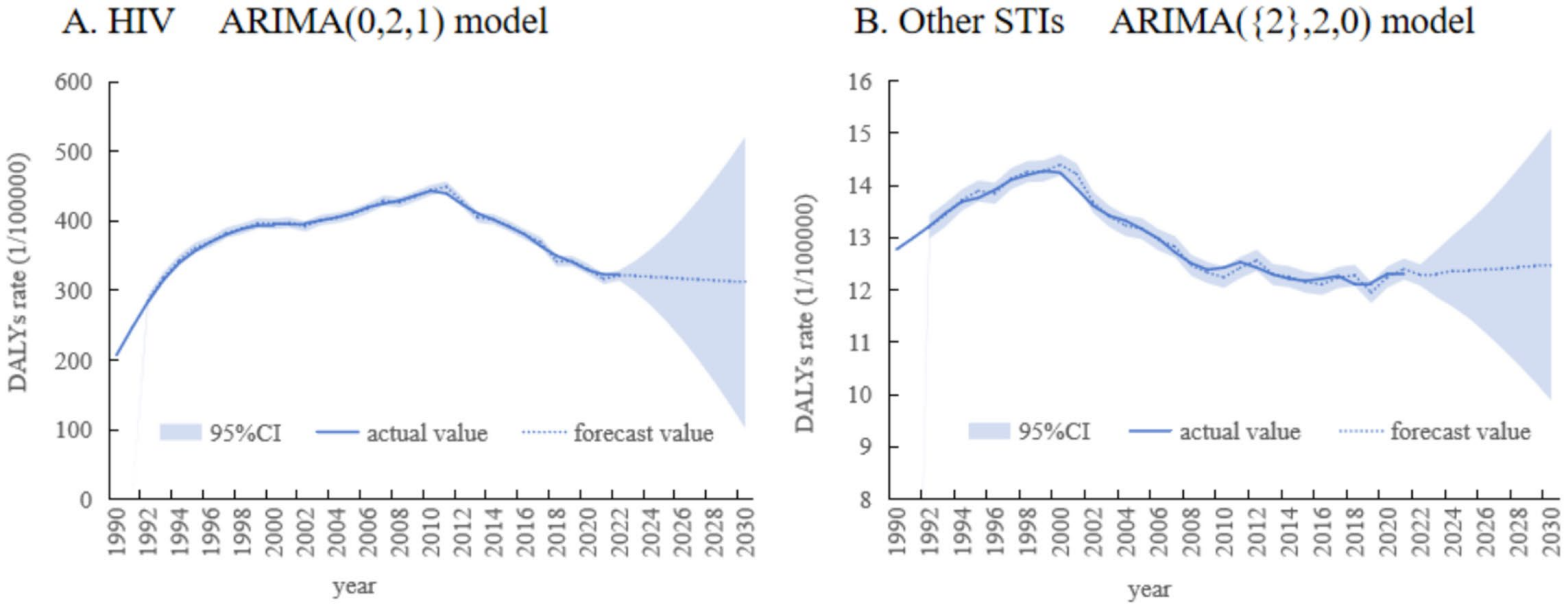
The DALYs rate per 100,000 people from 2022 to 2030 predicted by ARIMA. **(A)** The DALYs rate of HIV. **(B)** The DALYs rate of other STIs.

In this study, ARIMA model was used to predict the DALYs rate caused by AIDS (HIV) and other STIs among women of childbearing age worldwide from 2022 to 2030. Model parameters and prediction results are shown in Tables 4, 5 and Figure 5. It can be seen that in the next few years, the global HIV DALYs rate will continue to maintain a downward trend, while the DALYs rate of other STIs will increase. It is estimated that by 2030, the DALYs rate of HIV will be 311.68/100,000, and the DALYs rate of other STIs is 12.49/100,000.

**Table 4:** The goodness of fit metrics for ARIMA.

Disease	Model	AIC	BIC	MAPE (%)	RMSE (%)	Ljung-Box		Parameter	
						Q	*p*	Value	*p*
HIV	ARIMA (0,2,1)	166.24	167.64	0.75	3.74	1.86	0.868	−0.785	<0.0001
STIs	ARIMA ({2},2,0)	−49.84	−48.44	0.59	0.10	6.91	0.228	−0.436	0.013

**Table 5:** ARIMA prediction of DALYs.

Disease	2022	2023	2024	2025	2026	2027	2028	2029	2030
HIV	321.88	320.60	319.33	318.06	316.78	315.51	314.23	312.96	311.68
Other STIs	12.29	12.31	12.36	12.39	12.40	12.42	12.45	12.47	12.49

## Discussion

Among individuals living with HIV, 53% are women and girls (2). Due to the anatomical and physiological characteristics of the female reproductive tract, women are more biologically susceptible to infections than men. Women of reproductive age, being sexually active, are at heightened risk of HIV acquisition. Additionally, social determinants such as gender inequality, lower socioeconomic status, and limited healthcare access further exacerbate their vulnerability (5). In Rwanda, HIV prevalence among women (3.70%) was significantly higher than in men (2.20%) (6). In many sub-Saharan African countries, girls and women aged 15–24 were over three times more likely to contract HIV than their male counterpart (2). In the United States, women—particularly Black and Latino women facing racial disparities and economic hardship—were disproportionately affected by HIV (7). Furthermore, women living with HIV face the additional risk of mother-to-child transmission, with potentially severe consequences. Collectively, these biological, social, and economic factors pose substantial challenges for women following HIV infection.

### Development trends

Findings from this study indicate that global efforts have led to a decline in HIV incidence among women of reproductive age (15–49 years), with infection rates lower in 2021 than in 1990. Since 1997, HIV incidence has declined sharply, coinciding with the introduction of antiretroviral therapy (ART) in 1996 (8). After a stabilization phase between 2005 and 2015, incidence rates resumed a marked decline from 2015 onward, likely due to the implementation of targeted HIV prevention strategies for high-risk populations. In 2015, the World Health Organization (WHO) introduced pre-exposure prophylaxis (PrEP) as a preventive measure for high-risk groups, integrating it with other prevention strategies (9). Additionally, WHO issued guidelines advocating for the early initiation of HIV treatment to improve clinical outcomes. The trajectory of HIV-related DALYs followed a distinct pattern, increasing from 1990 to 2003 before declining from 2003 to 2021. This decline can be attributed to greater public awareness, increased investment in HIV management, and large-scale global health initiatives. In 2003, the U.S. Centers for Disease Control and Prevention (CDC) expanded its HIV response, and the President’s Emergency Plan for AIDS Relief (PEPFAR) was launched as the largest international health initiative by a single country dedicated to combating a single disease (10). PEPFAR has since invested over $100 billion in HIV prevention, care, and treatment across more than 50 countries. Over the past three decades, concerted global efforts have yielded substantial progress in addressing the HIV epidemic.

As far as the overall trend is concerned, from 1990 to 2021, the age-standardized incidence of HIV among women of childbearing age worldwide decreased by an average annual rate of 1.94%, while the age-standardized DALY rate of the same group increased by an average annual rate of 1.67% during the same period, showing a reverse trend. This reverse trend can be explained from two main lines “stock-increment dislocation” and “gender-age weighting.” First of all, the popularization of pregnancy and prenatal check-up, mother-to-child blockade and “treatment is prevention” have sharply reduced new infections. However, ART has pulled a large number of young mothers back from the death line, survived for a long time and included in the number of DALY every year, forming a stock accumulation of “reduced water inflow and higher water level.” Secondly, the GBD model gives higher disability weight to women aged 15–49, and superimposes gynecological comorbidities, menopausal aging, and social loss conversion of childbearing-parenting roles, so that the YLD contributed per unit surviving person-year is enlarged year by year. As a result, the incidence rate continues to decline in terms of “increment,” while the DALY rate increases in terms of “stock disability intensity,” and finally shows a statistical bell mouth in which the incidence rate decreases and the DALY rate increases at the same time.

The sharp increase in STI incidence observed in 2015 may be attributed to a series of measures implemented by the WHO to enhance STI surveillance and testing. That year, WHO emphasized the critical role of surveillance, encouraging countries to adopt standardized monitoring methods, assess STI burden and impact, and develop strategic information systems to map epidemiological trends and guide prevention efforts. Additionally, WHO issued guidelines recommending the use of point-of-care testing (POCT) technologies, particularly for dual HIV and syphilis screening, to improve detection rates. These initiatives aimed to enhance the accessibility and efficiency of STI diagnostics, ensuring that a greater number of patients received timely diagnoses and treatment. As testing methods improved and screening programs expanded, the detection of asymptomatic and previously overlooked cases increased, contributing to the observed rise in STI incidence (11, 12). The peak in STI incidence in 2019 was closely linked to disruptions caused by the COVID-19 pandemic. During this period, access to STI testing and counseling services was severely restricted as many healthcare facilities reduced or suspended non-essential services, including sexual health clinics. The resulting decline in routine STI screening not only increased the likelihood of undiagnosed and untreated infections but also compromised the accuracy of public health data. Many STI cases went unreported, significantly impacting sexual health services and potentially distorting epidemiological trends (13).

### Regional disparities

The findings highlight significant regional disparities in the burden of HIV among women of reproductive age. In 1990, the regions with the highest HIV burden among women of childbearing age in the world were concentrated in the Caribbean islands and sub-Saharan Africa (central, eastern, southern and western). The main ethnic groups in these regions, Afro-Caribbean and Black Africans in sub-Saharan Africa, are at the disadvantage of colonial history, racialized poverty and gender norms. In Africa, black women are locked in the most resource-scarce villages by colonial borders and structural adjustment. Polygamy, female genital mutilation, levirate inheritance and other ethnic internal norms make the virus spread rapidly along the family. In the 1980s, European and American media labeled HIV as the “African plague,” and international pharmaceutical companies delayed providing treatment, leaving the vast majority of infected black women with no medicine to take and becoming a community-level virus repository. It is this triple superposition of “ethnic group-history-economy” that makes the virus quickly take root and continue to enlarge among women of childbearing age. Racial and ethnic disparities are key determinants of health outcomes in these regions, providing a historical basis for today’s precise prevention and control strategies targeting different ethnic backgrounds. In 2021, the significant decline in AIDS incidence in the Caribbean and Africa is a positive signal, indicating that the prevention and treatment measures in recent years have achieved remarkable results.

In contrast, regions with historically low HIV incidence in 1990—such as Central Asia, Eastern Europe, and Oceania—experienced substantial increases by 2021. These shifts align with the historical trajectory of the global HIV epidemic. The origins of HIV can be traced to Central Africa in the early 20th century, with subsequent spread to Europe, Asia, and Oceania through African migration and transcontinental travel. While HIV reached Asia in the late 1980s, its incidence remained relatively low in the region until globalization and increased population mobility facilitated cross-border transmission. Many countries, particularly those with limited healthcare resources, struggled to manage imported HIV cases effectively, contributing to the significant rise in incidence in Central Asia, Eastern Europe, and Oceania by 2021. Notably, Oceania exhibited the most rapid growth in HIV incidence and DALYs, warranting focused attention.

Western Europe recorded the lowest age-standardized incidence of STIs, likely attributable to higher condom usage, greater access to sexual health education, and more favorable socioeconomic conditions.

### Specific STIs and HIV

There is a significant synergistic relationship between STIs and HIV. Both of them show the characteristics of mutual promotion and aggravation in biological mechanism, epidemiological characteristics and public health prevention and control14 (22). Biologically, STIs can destroy the genital mucosal barrier and increase the risk of HIV transmission. HIV infection weakens the immune system, making STIs more severe and more difficult to cure. In terms of epidemiological characteristics, studies have shown that the existence of STIs can increase the probability of HIV transmission by 2 ~ 3 times. On the contrary, HIV-infected people are also more susceptible to other STIs, forming an “epidemiological synergistic effect” and leading to a vicious circle. In terms of public health prevention and control, the public health systems of many countries manage HIV and STIs separately, resulting in redundant construction, missed opportunities for joint intervention, insufficient service coverage for high-risk groups, and limited prevention and control effects. Integrating HIV and STIs screening in the same service platform can improve the early detection rate, reduce the risk of transmission, optimize resource allocation, promote joint screening, intervention and policy formulation, and break the vicious circle of collaborative transmission to achieve more effective public health prevention and control goals.

Trichomoniasis accounted for the highest proportion of STIs included in this study. A systematic review and meta-analysis demonstrated that Trichomonas vaginalis infection increases the risk of HIV acquisition by approximately 50% (14). Individuals with T. vaginalis were found to have a 1.5-fold higher likelihood of contracting HIV compared to those without infection (14). Given the strong association between trichomoniasis and HIV, targeted screening and treatment for T. vaginalis could serve as an effective strategy to mitigate new HIV infections. The development and implementation of trichomoniasis control programs play a pivotal role in curbing HIV transmission.

Findings from this study indicate that trichomoniasis and chlamydia accounted for the majority of STI cases worldwide. Across all regions, the incidence of chlamydia and trichomoniasis among women surpassed that of gonorrhea and syphilis. Two key factors may contribute to this trend. First, approximately 85% of individuals infected with T. vaginalis remain asymptomatic (15), and chlamydia infections, particularly in women, often present with mild or no symptoms. As a result, a significant proportion of infections remain undiagnosed and untreated, leading to prolonged infection durations. Second, reinfection with trichomoniasis and chlamydia is common following the currently recommended single-dose treatment regimens, primarily due to inadequate treatment of sexual partners (16). Future research should prioritize the development of more effective screening and diagnostic approaches, enhancement of public awareness regarding chlamydia and trichomoniasis infections, and improvement of treatment adherence and efficacy.

Globally, the highest age-standardized incidence of syphilis was recorded in Africa. A survey revealed significant disparities in syphilis testing rates, with median screening coverage ranging from 83 to 99% in other regions but only reaching 58% in Africa (17). Expanding access to antenatal syphilis screening is essential for accurately assessing regional disparities in syphilis burden and formulating targeted public health strategies. Moreover, prenatal screening is critical for preventing congenital syphilis. In several African regions, syphilis screening implementation lags behind that of other infectious disease control efforts, necessitating further reinforcement of screening programs to reduce disease transmission and associated complications.

As illustrated in Figure 4, the age-standardized DALY rate for HIV significantly exceeds that of other STIs, underscoring its higher lethality and greater disease burden. Individuals living with HIV exhibit elevated morbidity and mortality across various non-AIDS-defining malignancies, including Hodgkin lymphoma, anal cancer, and hepatocellular carcinoma. Contributing factors such as population aging, chronic inflammation, persistent immune activation, and co-infections further drive non-AIDS-related mortality rates (18, 19).

### SDI regions

The analysis revealed an inverse relationship between the age-standardized incidence and DALY rates for HIV and other STIs among women of childbearing age and the SDI trend. A 30-year assessment of the AIDS disease burden corroborates these findings (20). This pattern highlights the persistent vulnerability of low-SDI regions, emphasizing the necessity for sustained improvements in sanitation infrastructure, universal healthcare access, and community-based educational initiatives to mitigate disease transmission. WHO estimates indicate that over 90% of new STI cases in 2016 occurred in low- and middle-income countries, likely driven by economic constraints, inadequate healthcare services, limited sexual health awareness, insufficient screening, and low treatment adherence (21).

### Age-specific trends

This study observed a “double peak” phenomenon with the highest age-standardized incidence of HIV in women aged 25–29 years and the highest DALY rate of STIs aged 30–34 years. This is the result of the superposition of multiple risk factors at this stage, such as enhanced biological susceptibility, gender power imbalance, transmission of online dating, and time cost of health services. These factors are amplified layer by layer in the life cycle of 25–34 years old, making the virus invade quickly in this age group first, and then accumulate into the heaviest disease burden. Therefore, only by shifting the intervention from a single age factor to multiple factors can the incidence rate and DALY peak be simultaneously reduced, and the maximum health benefits of the female group can be achieved.

### Forecast by ARIMA models

Time series analysis was used to scientifically predict the DALYs rate of HIV and other STIs in women of childbearing age in the next few years. The results show that the disease burden of HIV is on a downward trend, which may be due to the improvement of antiviral therapy (ART) coverage, the promotion of mother-to-child blocking measures, and the popularization of sexual health education in recent years. However, despite the overall decline, the absolute burden of HIV is still high, especially in some high-risk groups, and intervention measures still need to be continuously strengthened. In contrast, the DALYs rate of other STIs is on the rise. This trend suggests that the current prevention and control strategies for STIs may be insufficient, and early screening, partner tracking, health education and vaccine development need to be strengthened.

### Limitations

Despite its strengths, this study has certain limitations. First, reliance on the GBD database introduces potential inaccuracies due to variations in data sources across different countries and regions. Second, data quality and completeness, particularly in low- and middle-income nations, may have influenced the findings. Third, the absence of specific data collection and monitoring systems for HIV-positive pregnancies in existing databases precluded a more granular and precise analysis of global trends in HIV incidence during pregnancy. Fourth, the study only described the burden of HIV and five common STIs (syphilis, chlamydia, gonorrhea, trichomoniasis and genital herpes) among women of childbearing age, and did not include other STIs. Fifth, our research only discussed the direct influencing factors, such as policy, economy, medical care, etc., but there were many influencing factors, and they often interacted with each other, so we could not provide a comprehensive analysis.

## Conclusion

To the best of our knowledge, this is the first study to comprehensively examine the incidence and DALY trends of HIV and other STIs among women of childbearing age (15–49 years) from 1990 to 2021 at global, regional, and national levels. Based on GBD 2021, this analysis provides critical insights into the burden of HIV and other STIs across diverse populations and regions, informing the development of tailored prevention, screening, and treatment strategies.

## Data Availability

The original contributions presented in the study are included in the article/Supplementary material, further inquiries can be directed to the corresponding authors.
