# Supplementary material for: The trend analysis of HIV and other sexually transmitted infections among women of childbearing aged 15 to 49 years from 1990 to 2021 and its forecasting from 2022 to 2030
Source: Front Public Health. 2025 Dec 19;13:1702289. doi: 10.3389/fpubh.2025.1702289 (PMC12757296; doi:10.3389/fpubh.2025.1702289)
Supplement: Supplementary file 1 [file Supplementary_file_1.docx]

**Supplementary Files**

Table S1.Incident cases and age-standardized incidence rates of genital herpes among women of childbearing aged 15-49 years in 1990 and 2021 and average annual percentage change from 1990 to 2021...............................................................................................................................................................................................................................................................................................................................................................2

Table S2.Incident cases and age-standardized incidence rates of chlamydial infection among women of childbearing aged 15-49 years in 1990 and 2021 and average annual percentage change from 1990 to 2021.................................................................................................................................................................................................................................................................................................................................................................3

Table S3.Incident cases and age-standardized incidence rates of gonococcal infection among women of childbearing aged 15-49 years in 1990 and 2021 and average annual percentage change from 1990 to 2021..................................................................................................................................................................................................................................................................................................................................................................4

Table S4.Incident cases and age-standardized incidence rates of syphilis among women of childbearing aged 15-49 years in 1990 and 2021 and average annual percentage change from 1990 to 2021..................................................................................................................................................................................................................................................................................................................................................................5

Table S5.Incident cases and age-standardized incidence rates of trichomoniasis among women of childbearing aged 15-49 years in 1990 and 2021 and average annual percentage change from 1990 to 2021.................................................................................................................................................................................................................................................................................................................................................................6

| **Table S1.Incident cases and age-standardized incidence rates of genital herpes among women of childbearing aged 15-49 years in 1990 and 2021 and average annual percentage change from 1990 to 2021** | | | | | |
| --- | --- | --- | --- | --- | --- |
|  |  |  |  |  |  |
|  | Case（n，95%CI）,1990 | ASR per 100000 population（95%CI）,1990 | Case（n，95%CI）,2021 | ASR per 100000 population（95%CI）,2021 | AAPC(95%CI),1990-2021 |
| Global | 14631444(12415464 to 17017991) | 1061.7(1061.7 to 1061.7) | 22010116(18426849 to 25626423) | 1140.4(1140.4 to 1140.4) | 0.23(0.2 to 0.26) |
| Region |  |  |  |  |  |
| Andean Latin America | 217882(192901 to 243627) | 2104.3(2104 to 2104.6) | 379398(321077 to 436164) | 2147.2(2147 to 2147.4) | 0.07(0.04 to 0.1) |
| Australasia | 55489(46139 to 66246) | 1051(1050.7 to 1051.3) | 58674(47658 to 69666) | 850(849.8 to 850.2) | -0.66(-0.71 to -0.6) |
| Caribbean | 184371(155473 to 214053) | 1874.4(1874.1 to 1874.6) | 217894(182478 to 251079) | 1818.8(1818.6 to 1819.1) | -0.11(-0.14 to -0.08) |
| Central Asia | 128896(103879 to 154232) | 743.8(743.6 to 743.9) | 186163(150676 to 223409) | 748(747.9 to 748.1) | 0.02(0.01 to 0.03) |
| Central Europe | 157802(129435 to 187796) | 513.8(513.7 to 513.9) | 132538(105530 to 159530) | 517.9(517.8 to 517.9) | 0.03(0 to 0.06) |
| Central Latin America | 829536(719662 to 935471) | 1849(1848.8 to 1849.1) | 1223554(1029634 to 1412729) | 1802.1(1802 to 1802.2) | -0.08(-0.12 to -0.04) |
| Central Sub-Saharan Africa | 401763(352298 to 448516) | 2796(2795.7 to 2796.3) | 1032422(907976 to 1154732) | 2783(2782.8 to 2783.1) | -0.04(-0.06 to -0.02) |
| East Asia | 2941477(2421042 to 3521982) | 880.3(880.3 to 880.4) | 2948070(2395007 to 3538279) | 880.8(880.7 to 880.8) | 0(-0.05 to 0.04) |
| Eastern Europe | 583866(478043 to 703443) | 1048.1(1048 to 1048.2) | 469121(381674 to 561005) | 1048.8(1048.7 to 1048.9) | 0(0 to 0.01) |
| Eastern Sub-Saharan Africa | 982535(842889 to 1117978) | 1999(1998.9 to 1999.2) | 2338166(1970943 to 2695149) | 1968.8(1968.7 to 1968.9) | -0.05(-0.09 to -0.01) |
| High-income Asia Pacific | 479413(429391 to 535923) | 1077.7(1077.6 to 1077.8) | 313100(253845 to 376999) | 854.6(854.5 to 854.7) | -0.73(-0.78 to -0.69) |
| High-income North America | 903108(738946 to 1073329) | 1223.5(1223.4 to 1223.5) | 913312(749131 to 1078569) | 1112.7(1112.6 to 1112.8) | -0.31(-0.38 to -0.23) |
| North Africa and Middle East | 771402(643005 to 893636) | 945.1(945.1 to 945.2) | 1461932(1199337 to 1729376) | 918.6(918.5 to 918.6) | -0.09(-0.11 to -0.07) |
| Oceania | 21193(17396 to 24797) | 1285(1284.4 to 1285.5) | 47648(39261 to 56002) | 1333.6(1333.2 to 1333.9) | 0.12(0.06 to 0.17) |
| South Asia | 1428113(1163113 to 1705733) | 567.9(567.8 to 567.9) | 3002455(2451903 to 3595154) | 605.3(605.2 to 605.3) | 0.21(0.19 to 0.22) |
| Southeast Asia | 1425503(1215190 to 1651467) | 1130.8(1130.7 to 1130.8) | 1993421(1653886 to 2362742) | 1097.9(1097.8 to 1097.9) | -0.1(-0.12 to -0.07) |
| Southern Latin America | 219905(202716 to 236389) | 1745.7(1745.5 to 1746) | 294433(247718 to 341716) | 1722.1(1721.9 to 1722.3) | -0.04(-0.08 to 0) |
| Southern Sub-Saharan Africa | 358912(304745 to 409385) | 2438(2437.7 to 2438.2) | 569243(493299 to 642368) | 2568.1(2567.8 to 2568.3) | 0.18(0.15 to 0.21) |
| Tropical Latin America | 855257(725070 to 986549) | 2030.4(2030.2 to 2030.5) | 1215175(1030626 to 1394241) | 2034.2(2034.1 to 2034.3) | 0.01(-0.02 to 0.04) |
| Western Europe | 729927(605812 to 867328) | 768.6(768.5 to 768.6) | 657524(536722 to 780871) | 737.7(737.7 to 737.8) | -0.12(-0.16 to -0.07) |
| Western Sub-Saharan Africa | 955095(798850 to 1107967) | 1998.5(1998.4 to 1998.6) | 2555874(2143780 to 2970442) | 1986.1(1986.1 to 1986.2) | -0.03(-0.04 to -0.01) |

| **Table S2.Incident cases and age-standardized incidence rates of chlamydial infection among women of childbearing aged 15-49 years**  **in 1990 and 2021 and average annual percentage change from 1990 to 2021** | | | | | |
| --- | --- | --- | --- | --- | --- |
|  |  |  |  |  |  |
|  | Case（n，95%CI）,1990 | ASR per 100000 population（95%CI）,1990 | Case（n，95%CI）,2021 | ASR per 100000 population（95%CI）,2021 | AAPC(95%CI),1990-2021 |
| Global | 65012611(46242643 to 90559870) | 4931.8(4931.7 to 4931.8) | 99376268(70850219 to 140235666) | 5048.1(5048.1 to 5048.2) | 0.07(0.04 to 0.11) |
| Region |  |  |  |  |  |
| Andean Latin America | 528063(370757 to 725768) | 5650(5649.5 to 5650.4) | 968933(681038 to 1368139) | 5476.1(5475.7 to 5476.4) | -0.08(-0.33 to 0.18) |
| Australasia | 86240(61021 to 120757) | 1589.5(1589.1 to 1589.8) | 116904(81985 to 165165) | 1585.8(1585.6 to 1586.1) | 0(-0.12 to 0.13) |
| Caribbean | 864131(602421 to 1195494) | 9419.6(9418.9 to 9420.2) | 1142835(808847 to 1596108) | 9417.2(9416.6 to 9417.7) | 0.01(0 to 0.01) |
| Central Asia | 2125114(1484726 to 2982278) | 12521.7(12521.2 to 12522.3) | 3143193(2231320 to 4385098) | 12521.4(12520.9 to 12521.8) | 0(-0.01 to 0.01) |
| Central Europe | 1585894(1131413 to 2275915) | 5097.2(5096.9 to 5097.4) | 1342247(969954 to 1929890) | 5045.2(5044.9 to 5045.5) | -0.03(-0.04 to -0.03) |
| Central Latin America | 3609460(2575226 to 4899745) | 8882(8881.7 to 8882.3) | 6124618(4364771 to 8496146) | 8936.5(8936.3 to 8936.8) | 0.03(-0.03 to 0.08) |
| Central Sub-Saharan Africa | 334151(231482 to 472474) | 2799.3(2799 to 2799.6) | 867365(595608 to 1234972) | 2756.1(2755.9 to 2756.3) | -0.05(-0.06 to -0.04) |
| East Asia | 20171119(14206496 to 28796997) | 6313.8(6313.7 to 6313.9) | 24827495(17892318 to 36078368) | 6923.9(6923.8 to 6924) | 0.29(0.21 to 0.37) |
| Eastern Europe | 4327343(3054728 to 6306750) | 7362.7(7362.5 to 7362.9) | 3895460(2801058 to 5611172) | 7407.7(7407.5 to 7408) | 0.02(0 to 0.03) |
| Eastern Sub-Saharan Africa | 2269890(1612452 to 3071856) | 5365.4(5365.1 to 5365.6) | 5164182(3607472 to 7141024) | 4980.7(4980.5 to 4980.8) | -0.24(-0.34 to -0.14) |
| High-income Asia Pacific | 605745(432602 to 838624) | 1330.2(1330.1 to 1330.3) | 537708(385002 to 764040) | 1394.8(1394.7 to 1394.9) | 0.15(0.09 to 0.2) |
| High-income North America | 590820(406184 to 823874) | 778.6(778.5 to 778.6) | 798993(560087 to 1113610) | 946(946 to 946.1) | 0.61(0.51 to 0.71) |
| North Africa and Middle East | 5494345(4015432 to 7309584) | 7060.7(7060.5 to 7060.9) | 9907700(7076063 to 13632403) | 6189.6(6189.5 to 6189.8) | -0.41(-0.5 to -0.32) |
| Oceania | 237764(174681 to 313702) | 15223.8(15221.8 to 15225.8) | 454232(323530 to 629511) | 13034.8(13033.6 to 13036) | -0.49(-0.57 to -0.41) |
| South Asia | 6983369(4800056 to 9846007) | 2814.8(2814.7 to 2814.9) | 14308368(9949832 to 20289716) | 2895.8(2895.7 to 2895.8) | 0.08(-0.02 to 0.17) |
| Southeast Asia | 7167404(5104153 to 9899002) | 6133.2(6133.1 to 6133.4) | 11005381(7822462 to 15641434) | 5923.9(5923.8 to 5924) | -0.1(-0.15 to -0.05) |
| Southern Latin America | 154651(111968 to 212587) | 1254.2(1254 to 1254.4) | 232281(164528 to 330170) | 1313.3(1313.2 to 1313.5) | 0.15(0.14 to 0.17) |
| Southern Sub-Saharan Africa | 1366191(963609 to 1873240) | 10520.2(10519.6 to 10520.8) | 2175958(1544360 to 3036743) | 9872.9(9872.5 to 9873.4) | -0.2(-0.28 to -0.12) |
| Tropical Latin America | 3963291(2736062 to 5573951) | 10136.8(10136.5 to 10137.1) | 6370741(4525399 to 9015337) | 10204.7(10204.5 to 10205) | 0.02(-0.03 to 0.06) |
| Western Europe | 588162(415210 to 821924) | 610.1(610.1 to 610.2) | 551389(392690 to 762263) | 597.6(597.5 to 597.6) | -0.06(-0.09 to -0.03) |
| Western Sub-Saharan Africa | 1959463(1359726 to 2714549) | 4644(4643.8 to 4644.2) | 5440284(3781568 to 7576578) | 4721.2(4721.1 to 4721.3) | 0.06(-0.01 to 0.13) |

| **Table S3.Incident cases and age-standardized incidence rates of gonococcal infection among women of childbearing aged 15-49 years**  **in 1990 and 2021 and average annual percentage change from 1990 to 2021** | | | | | |
| --- | --- | --- | --- | --- | --- |
|  |  |  |  |  |  |
|  | Case（n，95%CI）,1990 | ASR per 100000 population  （95%CI）,1990 | Case（n，95%CI）,2021 | ASR per 100000 population（95%CI）,2021 | AAPC(95%CI),  1990-2021 |
| Global | 22055536(15762846 to 30066113) | 1524(1524 to 1524) | 25057474(18593079 to 32635931) | 1323.5(1323.5 to 1323.5) | -0.45(-0.51 to -0.4) |
| Region |  |  |  |  |  |
| Andean Latin America | 24094(16344 to 35646) | 229.7(229.6 to 229.8) | 36626(25675 to 53449) | 209.8(209.8 to 209.9) | -0.32(-0.39 to -0.24) |
| Australasia | 11034(8188 to 15033) | 212(211.8 to 212.1) | 12732(9760 to 17179) | 191.6(191.4 to 191.7) | -0.34(-0.4 to -0.27) |
| Caribbean | 163365(107728 to 255130) | 1566.1(1565.8 to 1566.3) | 177241(121202 to 270902) | 1505.1(1504.9 to 1505.3) | -0.13(-0.16 to -0.1) |
| Central Asia | 867686(565086 to 1351536) | 4616.9(4616.6 to 4617.2) | 898974(605779 to 1358427) | 3989.4(3989.2 to 3989.7) | -0.47(-0.49 to -0.45) |
| Central Europe | 1053219(774786 to 1466940) | 3649.9(3649.7 to 3650.2) | 676114(506643 to 913050) | 3275.5(3275.2 to 3275.7) | -0.35(-0.39 to -0.31) |
| Central Latin America | 555106(396058 to 757183) | 1157.9(1157.8 to 1158) | 708790(520382 to 936075) | 1051.4(1051.3 to 1051.5) | -0.32(-0.34 to -0.29) |
| Central Sub-Saharan Africa | 149821(96063 to 228165) | 1037.7(1037.6 to 1037.9) | 365639(237603 to 569534) | 979.8(979.7 to 979.9) | -0.17(-0.26 to -0.08) |
| East Asia | 7015117(4315008 to 10376909) | 1896.8(1896.7 to 1896.8) | 4350405(2880708 to 6323061) | 1582.1(1582.1 to 1582.2) | -0.58(-0.71 to -0.45) |
| Eastern Europe | 2077916(1410769 to 2931447) | 4018.5(4018.4 to 4018.7) | 1359685(930204 to 1931485) | 3565.7(3565.5 to 3565.9) | -0.38(-0.45 to -0.31) |
| Eastern Sub-Saharan Africa | 1164580(821113 to 1632797) | 2231.8(2231.7 to 2232) | 2669404(1875701 to 3746175) | 2113.9(2113.9 to 2114) | -0.2(-0.3 to -0.1) |
| High-income Asia Pacific | 209864(153193 to 279318) | 464.8(464.7 to 464.8) | 139706(105733 to 181476) | 406.3(406.2 to 406.4) | -0.45(-0.48 to -0.41) |
| High-income North America | 194084(131885 to 274439) | 281.3(281.3 to 281.4) | 216934(147980 to 306155) | 271(270.9 to 271) | -0.13(-0.48 to 0.22) |
| North Africa and Middle East | 1946814(1380163 to 2742520) | 2145.4(2145.3 to 2145.5) | 2760506(1977742 to 4003969) | 1765.6(1765.5 to 1765.6) | -0.61(-0.66 to -0.56) |
| Oceania | 129052(87532 to 183354) | 7277.2(7276 to 7278.5) | 295492(202913 to 426387) | 8089.2(8088.3 to 8090.1) | 0.34(0.32 to 0.37) |
| South Asia | 2643333(1739004 to 3806891) | 958.7(958.7 to 958.8) | 4190907(2843941 to 6110040) | 827.7(827.7 to 827.8) | -0.47(-0.57 to -0.37) |
| Southeast Asia | 794305(587226 to 1039203) | 608(607.9 to 608) | 1041426(786991 to 1358085) | 583.5(583.5 to 583.6) | -0.13(-0.14 to -0.12) |
| Southern Latin America | 54994(38702 to 81358) | 433.7(433.6 to 433.8) | 70295(49535 to 104191) | 422.2(422.1 to 422.2) | -0.11(-0.16 to -0.05) |
| Southern Sub-Saharan Africa | 1074217(743542 to 1460063) | 6954.5(6954.1 to 6955) | 1388121(966378 to 1893501) | 6387.9(6387.6 to 6388.2) | -0.3(-0.46 to -0.14) |
| Tropical Latin America | 700214(435357 to 1070113) | 1599.9(1599.8 to 1600) | 865557(560932 to 1286189) | 1535.8(1535.7 to 1535.9) | -0.13(-0.28 to 0.03) |
| Western Europe | 142460(108377 to 186929) | 153.6(153.6 to 153.6) | 123067(95385 to 162895) | 139.6(139.5 to 139.6) | -0.33(-0.43 to -0.22) |
| Western Sub-Saharan Africa | 1084260(769332 to 1489563) | 2071.2(2071.1 to 2071.4) | 2709851(1919520 to 3710045) | 1931.5(1931.5 to 1931.6) | -0.24(-0.26 to -0.21) |

| **Table S4.Incident cases and age-standardized incidence rates of syphilis among women of childbearing aged 15-49 years in 1990 and 2021 and average annual percentage change from 1990 to 2021** | | | | | |
| --- | --- | --- | --- | --- | --- |
|  |  |  |  |  |  |
|  | Case（n，95%CI）,1990 | ASR per 100000 population（95%CI）,1990 | Case（n，95%CI）,2021 | ASR per 100000 population（95%CI）,2021 | AAPC(95%CI),1990-2021 |
| Global | 3644611(2528551 to 4866997) | 260.5(260.5 to 260.5) | 5355440(3717326 to 7240281) | 279.2(279.2 to 279.2) | 0.2(0.13 to 0.27) |
| Region |  |  |  |  |  |
| Andean Latin America | 37870(25943 to 51116) | 367.9(367.8 to 368) | 63119(43523 to 85333) | 359.2(359.1 to 359.3) | -0.11(-0.22 to 0.01) |
| Australasia | 4866(3376 to 6556) | 91(90.9 to 91.1) | 6039(4199 to 8127) | 85.8(85.7 to 85.9) | -0.19(-0.24 to -0.15) |
| Caribbean | 33370(23856 to 44158) | 336.3(336.2 to 336.4) | 43792(30863 to 58625) | 366.5(366.4 to 366.6) | 0.38(0.29 to 0.47) |
| Central Asia | 8710(5988 to 11737) | 49(48.9 to 49) | 10829(7589 to 14562) | 45.3(45.3 to 45.3) | -0.27(-0.53 to -0.02) |
| Central Europe | 13900(9844 to 18721) | 46(45.9 to 46) | 10572(7544 to 14234) | 43.2(43.2 to 43.2) | -0.24(-0.4 to -0.09) |
| Central Latin America | 74931(51497 to 101583) | 169.4(169.4 to 169.4) | 102849(72296 to 138554) | 151.2(151.2 to 151.2) | -0.37(-0.49 to -0.26) |
| Central Sub-Saharan Africa | 175594(127006 to 233657) | 1332.1(1331.9 to 1332.3) | 396285(275396 to 536101) | 1142.4(1142.3 to 1142.5) | -0.6(-0.83 to -0.37) |
| East Asia | 334520(228289 to 458677) | 98.5(98.5 to 98.5) | 350512(245454 to 473097) | 109.4(109.4 to 109.4) | 0.26(-0.39 to 0.92) |
| Eastern Europe | 24503(16818 to 34102) | 44.1(44.1 to 44.1) | 18996(13191 to 25623) | 41.4(41.4 to 41.4) | -0.2(-0.38 to -0.01) |
| Eastern Sub-Saharan Africa | 573680(425020 to 731790) | 1201.1(1201 to 1201.2) | 918296(648635 to 1235092) | 799.1(799 to 799.1) | -1.33(-1.46 to -1.21) |
| High-income Asia Pacific | 51207(35953 to 69278) | 114.5(114.4 to 114.5) | 38953(27472 to 52173) | 108.8(108.8 to 108.9) | -0.14(-0.18 to -0.1) |
| High-income North America | 98314(68399 to 134704) | 132.1(132.1 to 132.2) | 102433(71882 to 139154) | 124.2(124.1 to 124.2) | -0.22(-0.33 to -0.1) |
| North Africa and Middle East | 117775(80561 to 163456) | 142.6(142.6 to 142.6) | 212477(146410 to 288694) | 134.4(134.3 to 134.4) | -0.21(-0.26 to -0.15) |
| Oceania | 12327(8594 to 16549) | 737.3(736.9 to 737.7) | 23763(16506 to 32288) | 664.9(664.6 to 665.2) | -0.36(-0.48 to -0.25) |
| South Asia | 981798(660461 to 1331976) | 369.1(369.1 to 369.2) | 1431200(983195 to 1949503) | 283.9(283.9 to 283.9) | -0.91(-1.09 to -0.73) |
| Southeast Asia | 294044(198556 to 404658) | 228.9(228.9 to 229) | 417387(288110 to 572393) | 231.1(231.1 to 231.2) | 0.06(-0.3 to 0.42) |
| Southern Latin America | 32761(23856 to 43929) | 260.8(260.7 to 260.9) | 47865(33558 to 64842) | 280.6(280.5 to 280.7) | 0.49(-0.19 to 1.17) |
| Southern Sub-Saharan Africa | 286477(202190 to 374509) | 1989.2(1989 to 1989.4) | 212347(144525 to 290354) | 969.1(968.9 to 969.2) | -2.31(-2.46 to -2.16) |
| Tropical Latin America | 143785(96204 to 196060) | 345.9(345.9 to 346) | 303423(219553 to 387109) | 513.3(513.2 to 513.3) | 1.27(0.98 to 1.57) |
| Western Europe | 42780(30230 to 58258) | 44.9(44.9 to 44.9) | 38736(27281 to 52990) | 43.9(43.9 to 44) | -0.05(-0.08 to -0.03) |
| Western Sub-Saharan Africa | 301399(210254 to 402265) | 638.7(638.6 to 638.8) | 605566(416603 to 821775) | 475(474.9 to 475) | -0.97(-1.05 to -0.89) |

| **Table S5.Incident cases and age-standardized incidence rates of trichomoniasis among women of childbearing aged 15-49 years in 1990 and 2021 and average annual percentage change from 1990 to 2021** | | | | | |
| --- | --- | --- | --- | --- | --- |
|  |  |  |  |  |  |
|  | Case（n，95%CI）,1990 | ASR per 100000 population（95%CI）,1990 | Case（n，95%CI）,2021 | ASR per 100000 population（95%CI）,2021 | AAPC(95%CI),1990-2021 |
| Global | 79843270(56019752 to 108610148) | 6272.4(6272.3 to 6272.4) | 134715354(95550981 to 185462520) | 6788(6787.9 to 6788) | 0.26(0.23 to 0.28) |
| Region |  |  |  |  |  |
| Andean Latin America | 548195(384040 to 749712) | 6355.4(6354.9 to 6356) | 1145366(789179 to 1572362) | 6500.6(6500.3 to 6501) | 0.07(0.04 to 0.1) |
| Australasia | 202816(140616 to 282029) | 3689.4(3688.9 to 3689.9) | 296532(208567 to 409305) | 3833.9(3833.4 to 3834.3) | 0.13(0.08 to 0.18) |
| Caribbean | 784393(561795 to 1070472) | 9049.7(9049 to 9050.3) | 1117770(789927 to 1523956) | 9150.7(9150.1 to 9151.2) | 0.02(-0.04 to 0.09) |
| Central Asia | 1214608(854192 to 1653309) | 7622.8(7622.3 to 7623.2) | 1962120(1391411 to 2677737) | 7725.9(7725.5 to 7726.2) | 0.04(0.03 to 0.06) |
| Central Europe | 1696658(1176839 to 2357834) | 5312.1(5311.9 to 5312.4) | 1585293(1107791 to 2259399) | 5499(5498.8 to 5499.3) | 0.15(0.13 to 0.17) |
| Central Latin America | 5261693(3688892 to 7197408) | 13990.8(13990.4 to 13991.2) | 9463678(6750975 to 13099515) | 13732.2(13732 to 13732.5) | -0.08(-0.22 to 0.06) |
| Central Sub-Saharan Africa | 924703(664175 to 1248139) | 8307.7(8307.1 to 8308.2) | 2507024(1801043 to 3378765) | 8451.9(8451.6 to 8452.3) | 0.06(0.04 to 0.07) |
| East Asia | 16216527(11277906 to 22664938) | 5237.1(5237 to 5237.2) | 18358187(12700484 to 26740035) | 4920.1(4920.1 to 4920.2) | -0.2(-0.27 to -0.13) |
| Eastern Europe | 2328882(1584437 to 3310105) | 3964.8(3964.6 to 3965) | 2196566(1496133 to 3196634) | 3920.7(3920.5 to 3920.9) | 0.02(-0.07 to 0.11) |
| Eastern Sub-Saharan Africa | 6276883(4572837 to 8363535) | 16177.7(16177.2 to 16178.1) | 15888707(11583052 to 21159751) | 16341.2(16340.9 to 16341.4) | 0.04(-0.01 to 0.09) |
| High-income Asia Pacific | 2161538(1515053 to 3079722) | 4640(4639.8 to 4640.2) | 1925759(1338205 to 2791856) | 4595.3(4595.1 to 4595.5) | -0.02(-0.05 to 0.01) |
| High-income North America | 7244770(5048743 to 10157718) | 9187.7(9187.5 to 9188) | 7933651(5519532 to 11298259) | 8960.1(8959.9 to 8960.3) | -0.09(-0.31 to 0.14) |
| North Africa and Middle East | 3155207(2225501 to 4254535) | 4463.6(4463.5 to 4463.8) | 7109423(5030586 to 9760380) | 4400.5(4400.3 to 4400.6) | -0.03(-0.35 to 0.29) |
| Oceania | 250060(180897 to 326658) | 17389.9(17387.7 to 17392.1) | 567324(405227 to 757309) | 16702.2(16700.8 to 16703.6) | -0.11(-0.37 to 0.16) |
| South Asia | 9152762(6374250 to 12612435) | 3892.5(3892.4 to 3892.6) | 19440412(13492546 to 27233883) | 4004.3(4004.3 to 4004.4) | 0.06(-0.04 to 0.15) |
| Southeast Asia | 7115405(5010416 to 9753472) | 6455.7(6455.6 to 6455.9) | 11594950(8191747 to 16201494) | 6166.5(6166.3 to 6166.6) | -0.05(-0.14 to 0.04) |
| Southern Latin America | 497735(347459 to 681585) | 4094.3(4094 to 4094.7) | 763627(534568 to 1055815) | 4228(4227.7 to 4228.3) | 0.1(0.02 to 0.18) |
| Southern Sub-Saharan Africa | 2887255(2108988 to 3893264) | 23949.1(23948.3 to 23950) | 4742514(3383381 to 6461012) | 21664.7(21664.1 to 21665.3) | -0.33(-0.48 to -0.18) |
| Tropical Latin America | 3870617(2713611 to 5295724) | 10413.5(10413.1 to 10413.8) | 6614536(4653964 to 9492649) | 10297(10296.8 to 10297.3) | -0.03(-0.07 to 0.01) |
| Western Europe | 2196817(1543160 to 3032911) | 2227.9(2227.8 to 2228) | 2255474(1568358 to 3192053) | 2245.8(2245.7 to 2245.9) | 0.04(-0.06 to 0.15) |
| Western Sub-Saharan Africa | 5855746(4220916 to 7852591) | 15003.3(15002.9 to 15003.7) | 17246442(12293980 to 23187227) | 15949.2(15949 to 15949.5) | 0.17(0.08 to 0.26) |
